# Clinical application of a multiplex genetic pathogen detection system remaps the aetiology of diarrhoeal infections in Shanghai

**DOI:** 10.1186/s13099-018-0264-7

**Published:** 2018-09-11

**Authors:** Shiwen Wang, Feng Yang, Dong Li, Juanxiu Qin, Weiwei Hou, Lian Jiang, Mimi Kong, Yong Wu, Yuchen Zhang, Fuju Zhao, Yi Fang, Yingxin Miao, Lingli Xu, Jie Chen, Zhijun Bao, Michal A. Olszewski, Hu Zhao, Yanmei Zhang

**Affiliations:** 1 Department of Laboratory Medicine, Huadong Hospital, Affiliated with Fudan University, Shanghai, 200040 China; 2 Shanghai Key Laboratory of Clinical Geriatric Medicine, Shanghai, 200040 China; 3 Research Center on Aging and Medicine, Fudan University, Shanghai, 200040 China; 4 Department of Clinical Laboratory, Shanghai Tongji Hospital, Tongji University School of Medicine, Shanghai, 200065 China; 5 Department of Laboratory Medicine, Renji Hospital, School of Medicine, Shanghai Jiaotong University, Shanghai, 200135 China; 6 Ningbo HEALTH Gene Technologies Co., Ltd., Ningbo, China; 7 Shanghai ABSciex Analytical Instrument Trading Co., Ltd., Shanghai, China; 8 Department of Gastroenterology, Gerontology Institute of Shanghai, Affiliated with Huadong Hospital, Affiliated with Fudan University, Shanghai, 200040 China; 9 Division of Pulmonary and Critical Care Medicine, Department of Internal Medicine, University of Michigan Health System and Research Service, VA Ann Arbor Health Systems, Ann Arbor, MI USA

**Keywords:** Diarrhoeal pathogens (DPs), Rapid screening, High-throughput multiplex genetic detection system (HMGS), Faecal specimens, DP composition, Polymicrobial infection

## Abstract

**Background:**

Culture-based diagnostic methods cannot achieve rapid and precise diagnoses for the identification of multiple diarrhoeal pathogens (DPs). A high-throughput multiplex genetic detection system (HMGS) was adapted and evaluated for the simultaneous identification and differentiation of infectious DPs and a broad analysis of DP infection aetiology.

**Results:**

DP-HMGS was highly sensitive and specific for DP detection compared with culture-based techniques and was similar to singleplex real-time PCR. The uniform level of sensitivity of DP-HMGS for all DPs allowed us to remap the aetiology of acute diarrhoeal infections in Shanghai, correcting incidences of massively underdiagnosed DP species with accuracy approaching that of sequencing-based methods. The most frequent DPs were enteropathogenic *Escherichia coli*, rotavirus and *Campylobacter jejuni*. DP-HMGS detected two additional causes of infectious diarrhoea that were previously missed by routine culture-based methods: enterohemorrhagic *E. coli* and *Yersinia enterocolitica*. We demonstrated the age dependence of specific DP distributions, especially the distributions of rotavirus, intestinal adenovirus and *Clostridium difficile* in paediatric patients as well as those of dominant bacterial infections in adults, with a distinct “top 3” pattern for each age group. Finally, the multiplexing capability and high sensitivity of DP-HMGS allowed the detection of infections co-induced by multiple pathogens (approximately 1/3 of the cases), with some DPs preferentially co-occurring as infectious agents.

**Conclusions:**

DP-HMGS has been shown to be a rapid, specific, sensitive and appropriate method for the simultaneous screening/detection of polymicrobial DP infections in faecal specimens. Widespread use of DP-HMGS is likely to advance routine diagnostic and clinical studies on the aetiology of acute diarrhoea.

**Electronic supplementary material:**

The online version of this article (10.1186/s13099-018-0264-7) contains supplementary material, which is available to authorized users.

## Background

Diarrhoeal diseases caused by enteric infections continue to pose a major threat to global health [1, 2]. With respect to the overall impact on human health, diarrhoea ranks second among all infectious diseases [3, 4], with approximately 2 billion incidences of diarrhoeal diseases reported annually in China alone [5]. The World Health Organization (WHO)’s Global Burden of Disease study lists diarrhoeal diseases as one of the leading causes of preventable deaths worldwide [6].

One of the greatest challenges in the diagnosis of diarrhoeal diseases is that a large number of aetiological agents are associated with generally non-specific clinical symptoms [7]. Traditional detection methods for these pathogens in faecal specimens are culture-based methods, immunological detection assays, and molecular diagnostic methods [8]. The culture-based methods, while considered the “gold standard” options for routine diagnosis, are time consuming [9, 10]. Furthermore, infections caused by microbes with very stringent/specific requirements for culture conditions are likely to be underdiagnosed, especially in cases of polymicrobial infections. The development of molecular diagnostic methods such as real-time PCR, has increased the sensitivity of these methods, but typically, such testing is restricted to a single pathogen per test. Finally, metagenomic approaches using next-generation sequencing, while capable of generating virtually infinite amounts of information, require sophisticated analytical tools and time-consuming analysis and are very costly; thus, these methods cannot be broadly applied in everyday diagnosis of common diarrhoeas. Due to these limitations, precise aetiological diagnosis of enteric infections is not routinely accomplished, resulting in poor therapeutic efficacy and increased risks for the development of drug-resistant bacteria and introduction of imbalance in the intestinal microbiota (dysbiosis) [11, 12].

Studies conducted worldwide demonstrate wide variations in the prevalence and composition of causative agents of acute diarrhoea [13–16]. These discrepancies can arise due to non-uniform diagnostic approaches and preferential detection of some pathogens. These broad concerns resulted in the publication of clinical guidelines by the Expert Consensus on Diagnosis and Treatment of Infectious Diarrhoea in Chinese Adults (2013), which recommend aetiological diagnosis to promote rational treatment of diarrhoeal diseases, proper epidemiological studies of these diseases, and prevention of antimicrobial drug resistance [17, 18].

Here, we redesign and adapt a high-throughput multiplex genetic detection system (HMGS) to screening faecal specimens for 19 major pathogenic diarrhoeal pathogens (DPs) that cause acute diarrhoeal infections [19]. A total of 613 faecal specimens were analysed by the DP-HMGS assay, sequencing, and conventional methods (culture-based methods and singleplex real-time PCR) in parallel, and the methods were compared for accuracy and applicability in the generation of epidemiological data.

## Methods

### Ethics statement

This study was carried out in accordance with the recommendations of the Ethics Committee for Human Studies of Huadong Hospital and registered under Ethics Approval Number 2013-077 with written informed consent from all subjects. All subjects provided written informed consent in accordance with the Declaration of Helsinki.

### Diarrhoeal pathogens

Based on epidemiological investigations, the DPs that most commonly cause diarrhoeal diseases in the Shanghai area were selected as candidates for the DP-HMGS screening assay [20–22]. The six most common viral pathogens that cause outbreaks of gastroenteritis, namely, human astrovirus (HASV), norovirus II (NorV), human adenovirus (HADV), rotavirus A (RoVA), rotavirus B (RoVB), and rotavirus C (RoVC), as well as a negative control sapovirus were isolated from clinical specimens at Huadong Hospital, Shanghai, China and verified by sequencing of species-specific, conserved genes. A group of bacterial species that either most commonly cause enteritis and/or induce severe forms of enteritis were obtained from Shanghai Municipal Center for Disease Control & Prevention (CDC). Standard ATCC strains of the following bacterial species were used: *Campylobacter jejuni* (*C. jejuni*) ATCC33560*, Shigella* ATCC12022, pathogenic *Clostridium difficile* (*C. difficile*) ATCC9689*, Salmonella enteritidis* (*S. enteritidis*) ATCC31194*, Salmonella typhimurium* (*S. typhimurium*) ATCC14028*, Vibrio parahaemolyticus* (*V. parahaemolyticus*) ATCC17802*, Yersinia enterocolitica* (*Y. enterocolitica*) ATCC23715. From the CDC, we obtained the standard control microbes, namely, *Helicobacter pylori* (*H. pylori*) ATCC43504*, Pseudomonas aeruginosa* ATCC27853*, Staphylococcus aureus* (*S. aureus*) ATCC29213, and *Escherichia coli* (*E. coli*) ATCC35218. Other common bacterial DP strains selected for DP-HMGS testing, including 6 major pathogenic strains of *E. coli*—enterotoxigenic *E. coli* (ETEC), enterohemorrhagic *E. coli* (EHEC), enteropathogenic *E. coli* (EPEC), enteroaggregative *E. coli* (EAEC), enteroinvasive *E. coli* (EIEC), and *E. coli* O157 strain (*E. coli* O157)—as well as the non-pathogenic *E. coli* strain DH5α (*E. coli* DH5α) and *Plesiomonas shigelloides* were isolated from clinical specimens at Huadong Hospital, Shanghai, China, and verified by sequencing of species-specific conserved genes.

### Faecal specimen collection

Six hundred and thirteen faecal specimens were obtained from outpatients diagnosed with diarrhoea from January 2016 to November 2017, and 30 faecal specimens were obtained from healthy volunteers from Renji Hospital and Children’s Hospital, affiliated with Shanghai Jiaotong University; Tongji Hospital, affiliated with Tongji University; and the Centers for Disease Control in Songjiang district in Shanghai. Patients of all ages with symptoms of acute diarrhoea were considered to be eligible for enrolment. As per the ACG clinical guidelines, acute diarrhoea was defined as the occurrence of defecation 3 or more times per 24 h, with abnormal faecal characteristics, such as loose stool, watery stool, mushy stool, mucosal stool and bloody stool, lasting for less than 14 days [23]. The exclusion criteria were diarrhoea caused by medicines, poisons, food allergies food intolerance or other diseases. Patients undergoing antibiotic treatments were also excluded. Fresh whole faecal specimens (10 g) were collected in sterilized containers containing 2 mL of normal saline supplemented with recombinant RNase inhibitor (TaKaRa, Japan) to prevent degradation of genetic material from RNA viruses and stored at − 20 °C within 2 h.

### Nucleic acid extraction from faecal specimens

Total nucleic acid was extracted from a 200 μL faecal suspension using the Whole Genome Extraction Kit (Zhongding Biotech Co., Ltd., Ningbo, China) according to the manufacturer’s instructions. The extracts were eluted with 100 μL of DNase/RNase-free H_2_O (ddH_2_O). The concentrations of each extract were determined using a Thermo Nanodrop 2000 spectrophotometer (Thermo Fisher Scientific Inc., Waltham, MA, USA). The extracts were stored at − 80 °C until further analysis.

### Cloning and sequencing

The genomic targets of the selected pathogens were amplified, and the resulting products were purified using the High Pure PCR Product Purification Kit (Roche, Basel, Switzerland) and subsequently ligated into the pMD18-T simple vector. The constructs were transformed into *E. coli* DH5α, followed by sequencing by Shanghai RuiDi Biological Technology Company. Sequencing was performed using the Sanger method with an ABI 3730XL automated DNA analyser (Applied Biosystems Inc., California, USA). The DNA sequences were verified by a BLAST search of the National Center of Biotechnology Information (NCBI) nucleotide database (http://www.ncbi.nlm.nih.gov/blast) using DNASTAR Lasergene analysis software (DNASTAR Inc., WI, USA).

### Bacterial culture and identification

Faecal specimen suspensions were mixed briefly and transferred (1 mL) into a TissueLyser to obtain a uniform suspension (Jingxin Co., Ltd., Shanghai, China) and cultured with *Salmonella*-*Shigella* (SS) agar and Columbia blood agar at 37 °C for 24 h for *Shigella* and *S. typhimurium*. *C. jejuni* were cultured with charcoal cefoperazone deoxycholate agar (CCDA) under microaerophilic conditions at 37 °C for 24 h. The specimens were also inoculated into selective enrichment broth at 37 °C for 24 h, followed by subculturing on thiosulphate-citrate-bile salts-sucrose (TCBS) agar for culturing *Vibrio* species. Colonies of *Vibrio parahaemolyticus* and *Vibrio minicus* (green colonies on TCBS) and *Vibrio cholerae* and *Vibrio fluvialis* (yellow colonies on TCBS) were identified by matrix-assisted laser desorption/ionization time-of-flight mass spectrometry (BioMerieux, Lyon, France). Different serotypes of *E. coli* were inoculated onto Columbia blood agar and cultured at 37 °C for 24 h. *Y. enterocolitica* was inoculated into MacConkey agar and cultured at 28 °C for 48 h. *C. difficile* was inoculated into cycloserine cefoxitin fructose agar (CCFA) under anaerobic conditions a 37 °C for 24 h.

### Viral identification by singleplex real-time PCR

The Real-Time PCR Kit (BioPerfectus Technologies, Taizhou, China) was used to detect adenovirus, which is a DNA virus. Three reverse transcription PCR kits (BioPerfectus Technologies, Taizhou, China; including reverse transcriptase) were used to detect the RNA viruses norovirus, rotavirus and astrovirus. Four singleplex PCRs were conducted in a real-time PCR system (7500 real-time PCR system; ABI, California, USA) with software version 2.3. All procedures were conducted following the manufacturer’s instructions.

### Primer design

The 22 pairs of primers targeting the species-specific conserved genomic fragments of the selected DPs were designed to include 13 bacterial DPs (listed above), 6 viral DP (listed above), a human internal RNA control gene (hum_RNA) beta-2 microglobulin (*B2* *M*), a human internal DNA control gene (hum_DNA) ribonuclease P (*RNaseP*), and a systematic internal control (IC). Hundreds of sequences were downloaded from NCBI and analysed using Vector NTI to identify the most highly conserved gene targets specific for each individual DP type. The primers for amplification of the highly conserved regions were designed using DNASTAR software (DNASTAR Inc., Madison, WI, USA) and Primer Premier 5.0 software (Premier Biosoft International, Palo Alto, CA, USA). All the primers were synthesized and purified by Invitrogen™, China. These gene-specific primers were designed and optimized by applying the following criteria: homogeneity of primer sequences; amplification product sizes ranging from 100 to 350 bp, with at least 3-base-pair size differences between each fragment; absence of significant dimer formation between different primers; and absence of non-specific products with each pair of gene-specific primers.

The specific primer sets used in the DP-HMGS molecular detection assay and the corresponding amplicon sizes are all listed in Additional file 1: Table S1. The specificity of each single pair of primers was verified by singleplex PCR using templates containing all the corresponding extracted nucleic acids from each DP, and confirmed by Sanger sequencing. All the primers used for Sanger sequencing are listed in Additional file 2: Table S2. The primer pairs that generated amplification products with a single specific DP-HMGS peak but no non-specific peaks were selected (shown in Additional file 3: Figure S1A–Q).

### Setup of the DP-HMGS assay

Each DP-HMGS reaction contained 2 µL of 5× PCR buffer, 0.35 µL of 10 µM dNTPs, 0.25 µL of 25 mM MgCl_2_, 0.4 µL of 5 U/µL enzyme mix (Taq polymerase and reverse transcriptase), 0.1 µL of 1 U/µL anti-contamination enzyme UDG (uracil DNA glycosylase; TaKaRa, Japan) [24], 1 µM each of the forward and reverse primers, and 2.5 µL of plasmid; the amount of plasmid template for each target pathogen in the HMGS assay ranged from 5 to 50 ng. ddH_2_O was added to the PCR to attain a final volume of 10 µL. The PCR mixture was incubated as follows: 25 °C for 5 min; 50 °C for 30 min; 95 °C for 15 min; 35 cycles of 94 °C for 30 s, 60 °C for 30 s, and 72 °C for 1 min; 72 °C for 15 min.

### Separation by capillary electrophoresis and fragment analysis

Following the amplification step, 1 µL of the reaction product was added to 9 µL of highly deionized (Hi-Di) formamide along with 0.25 µL of DNA Size Standard 500 (AB Sciex, USA). The Applied Biosystems 3500DX genetic analysis system (Applied Biosystems, California, USA) was then used to analyse the PCR products based on size separation using high-resolution capillary gel electrophoresis. The peak height for each PCR product was reported in the electropherogram, and the reaction was considered to be positive when the dye signal was greater than 300 relative fluorescence units (rfu). ddH_2_O was used as a negative control throughout the experimental process.

### Establishment and optimization of the DP-HMGS assay

Multiple sets of primers and reaction parameters were used to optimize the performance of the DP-HMGS assay in a single reaction. The main optimization principle was to keep all amplicons that had similar amplification efficiency ranges and exhibited the gene-specific target amplicons. Primer sequences, concentrations and ratios were optimized so that each DP signature could be amplified specifically without cross-interaction. Additionally, the annealing temperature was optimized using the temperature gradient descent method (using chimeric primers, with temperatures from 50 to 65 °C). Other reaction parameters, such as buffer, enzyme, and reaction time, were also systematically optimized. The primers for the human internal RNA control gene *B2* *M* and human internal DNA control gene *RNaseP* were included in the DP-HMGS PCR primer mix. Detections of these two genes in the samples indicated that no significant nucleic acid degradation had occurred during specimen handling/storage. Additionally, a modified fragment of the kanamycin resistance gene (*Kanr*) was inserted into the pcDNA3.1 vector to generate a fusion plasmid that served as internal control for the detection system. The fusion plasmid (1.5 × 10^5^ copies in 3 µL) was added to the 200 µL faecal suspension, immediately prior to nucleic acid extraction, to monitor the extraction and the DP-HMGS reaction. The appearance of all 3 internal control peaks in the DP-HMGS trace confirmed that the sample RNA and DNA had good integrity and underwent efficient extraction, processing, and amplification.

### Sensitivity, specificity and accuracy of the DP-HMGS assay

The sensitivity of the DP-HMGS assay for each pathogen was tested by serial tenfold dilutions of plasmids. Serial tenfold dilutions of 22 plasmids using equal amounts of templates were used to test the simultaneous detection limit of the DP-HMGS for all pathogens. The specificity of the DP-HMGS assay in detecting pathogens in a microbiologically diverse gastrointestinal environment was tested using plasmids from 19 positive DPs from our panel combined with DNA from 7 negative control pathogen species expected to be present in the GI tract, namely, *H. pylori, E. coli* DH5α*, Pseudomona aeruginosa, S. aureus, Plesiomonas shigelloides, E. coli* and sapovirus (Additional file 4: Figure S2). To assess the accuracy, different amounts of three pathogen-associated plasmids (*S. enteritidis*, 1 × 10^3^ copies; HADV, 1 × 10^5^ copies; EHEC, 1 × 10^4^ copies) were randomly selected from the 19 types of DPs and mixed for testing with the DP-HMGS assay, and the results were compared with those of the single-template HMGS assay. The plasmids containing genes of the 7 negative control species were then mixed with plasmids containing genes from selected pathogenic species (*S. enteritidis*, HADV and EHEC) to further test the ability of the DP-HMGS assay to identify polymicrobial infections in microbiologically diverse environments. The reaction system setup and detection were performed as described above.

### Data analysis and statistics

The sensitivity and specificity of the diagnostic tests were calculated according to the following formulas: SE = TP/(TP + FN) × 100; SP = TN/(TN + FP) × 100; the positive predictive value (PPV) and negative predictive value (NPV) were calculated as follows: PPV = TP/P; NPV = TN/N (FN: false negative; FP: false positive; N: negative; P: positive; SE: Sensitivity; SP: specificity; TN: true negative; TP: true positive). Among these variables, TP refers to the number of samples that were positively detected by conventional methods (culture-based methods and singleplex real-time PCR) or DP-HMGS and the Sanger sequencing method. TN refers to the number of samples that gave negative results with conventional methods (culture-based methods and singleplex real-time PCR) or DP-HMGS and the Sanger sequencing method. FP refers to the number of samples that were positively detected by conventional methods (culture-based methods and singleplex real-time PCR) or DP-HMGS but gave negative results with the Sanger sequencing method. FN refers to the number of samples that gave negative results with conventional methods (culture-based methods and singleplex real-time PCR) or DP-HMGS but were positively detected by the Sanger sequencing method. The data were statistically analysed by the χ^2^ test using the Stata statistical software package, version 12.0 (Stata Corp College Station, TX, USA). The DP distribution of different groups was analysed by the Mann–Whitney rank-sum test for two variables and the Kruskal–Wallis H test for more than two variables. All of the above hypothesis tests were two-sided, and a two-tailed p-value of 0.05 or less was considered to indicate statistical significance.

## Results

### The optimized DP-HMGS assay allows for concurrent amplification of the DNA signature and simultaneous detection of up to 19 DPs in a single multiplex reaction

**Fig. 1:**
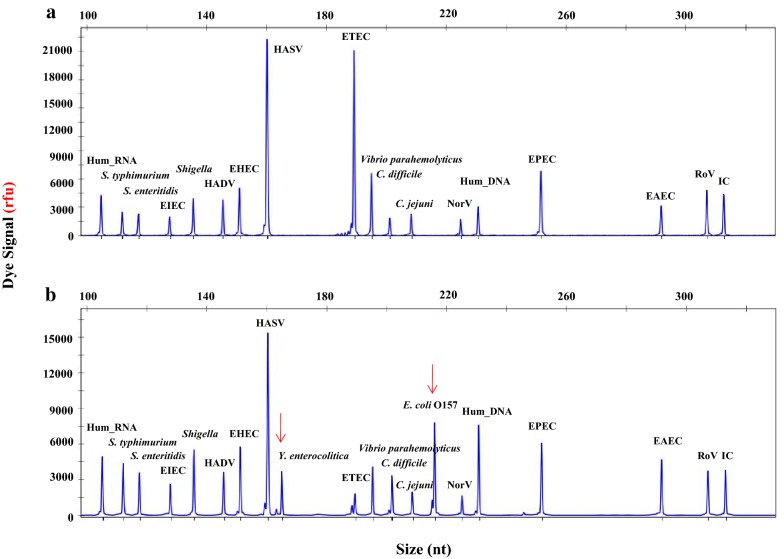
DP-HMGS was optimized for robust detection of DPs in a multiplex reaction. **a** Before optimization of the DP-HMGS assay, eighteen targets (from left to right: hum_RNA, *S. typhimurium, S. enteritidis,* EIEC*, Shigella,* HADV, EHEC, HASV, ETEC*, Vibrio, C. difficile, C. jejuni,* norovirus, hum_DNA, EPEC, EAEC, rotavirus, IC) exhibited relatively large differences in signal intensity. Notably, the specific peaks for *Y. enterocolitica* and *E. coli* O157 were not detected by the DP-HMGS assay prior to optimization. **b** After optimization, all 20 targets (from left to right: hum_RNA, *S. typhimurium, S. enteritidis,* EIEC*, Shigella,* HADV, EHEC, HASV, *Y. enterocolitica,* ETEC*, Vibrio, C. difficile, C. jejuni, E. coli* O157, norovirus, hum_DNA, EPEC, EAEC, rotavirus, IC) reached intermediate signal levels. Notably, the specific peaks for *Y. enterocolitica* and *E. coli* O157 were now visible at 165 bp and 218 bp

The 19 pairs of microbe-specific primers were designed for detection of the genetic signatures of these primers, and 3 primer sets were designed for quality control, as described in the Methods section (Additional file 1: Table S1). Following the testing of individual primer pairs for the detection of specific DP DNA signatures (Additional file 3: Figure S1), we evaluated the performance of all the primers combined in a single multiplex PCR (Fig. 1a). While most of the DP target signals were detected, substantial differences among the amplification signal intensities were initially observed; in addition, peaks for *Y. enterocolitica* and *E. coli* O157, the DNA templates for which were included in the 22-plex assay, were absent. To offset these differences in the amplification efficiencies of individual templates, the primer sequences and reaction parameters were further adjusted. This optimization process enabled us to detect all the pathogenic signatures (Fig. 1b) and to generate peaks for all the DNA targets with intermediate signal levels, which allowed for a potential increase or decrease in the signal based on variations among individual samples. Thus, a single multiplex-PCR-based DP screening assay (DP-HMGS) was developed and optimized for simultaneous detection of 19 major DPs with relatively uniform sensitivity.

### The DP-HMGS assay is highly specific and sensitive for the detection of DP signatures

To exclude the possibility of FP detection, nineteen pathogen templates and 3 controls were used as positive controls to test the specificity of the DP-HMGS assay, which produced specific amplification signals for the signature DNA from each target pathogen (Additional file 4: Figure S2A). This result was confirmed using DNA from 7 microbial species for which specific primers were not included in our DP-HMGS screening (*H. pylori*, *S. aureus*, *Pseudomonas aeruginosa*, *Plesiomonas shigelloides*, *E. coli* DH5α, *E. coli*, and sapovirus); the controls did not exhibit any specific amplification peaks, similar to the negative control ddH_2_O (Additional file 4: Figure S2B–I).

**Fig. 2:**
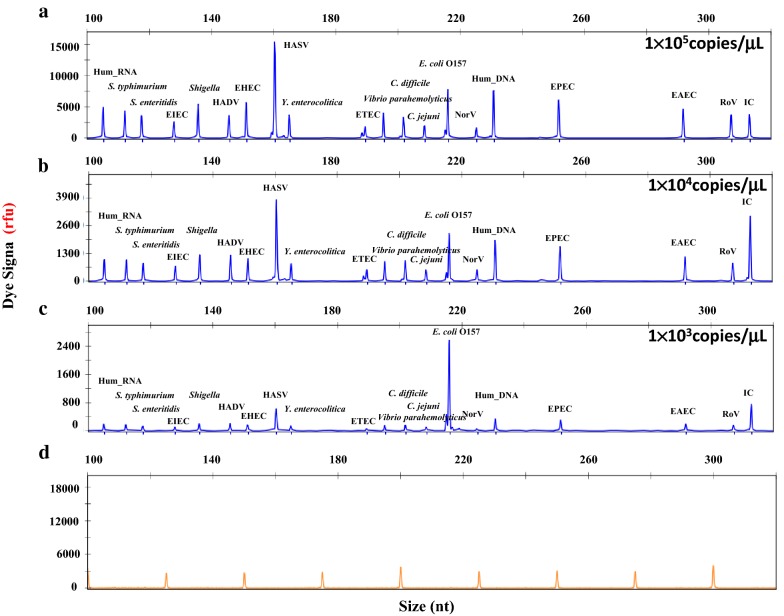
Optimized DP-HMGS maintained high sensitivity for the simultaneous detection of all specific viral and bacterial pathogens. The detection limits of the DP-HMGS assay were determined by amplifying tenfold diluted plasmids containing equal amounts of 17 pathogen and 3 quality control templates at **a** 1 × 10^5^, **b** 1 × 10^4^, and **c** 1 × 10^3^ copies/μL in the DP-HMGS assay. **d** ddH_2_O was used as the negative control. Note that the pathogen-defining DNA targets (from left to right: *S. typhimurium, S. enteritidis,* EIEC*, Shigella,* HADV, EHEC, HASV, *Y. enterocolitica,* ETEC*, Vibrio, C. difficile, C. jejuni, E. coli* O157, norovirus, EPEC, EAEC, rotavirus) all generated specific peaks. The quality controls Hum_RNA, Hum_DNA and IC produced specific peaks at 106 bp, 233 bp and 313 bp, respectively

Next, to determine the limit of detection in terms of copy number of the DP signature gene, we tested the sensitivity of DP-HMGS using various concentration ranges of the specific pathogen templates. Twenty-two plasmid pairs to amplify the DP target and control genes were generated and tested using a broad range of known concentrations. The assay was extremely reliable in the detection of 10^3^–10^5^ copies of all the microbial genes, demonstrating that the sensitivity for simultaneous detection of all pathogens by the DP-HMGS assay was at least 1 × 10^3^ copies/μL (Fig. 2), while further titration to 1 × 10^2^ gene copies/μL resulted in the generation of relatively small peaks, which for some pathogens were below the cut-off fluorescence signal of 300 rfu. Thus, reliable detection of specific DP signatures by DP-HMGS can consistently occur at a concentration of 1 × 10^3^ copies/μL.

### DP-HMGS can accurately detect individual DPs in polymicrobial mixtures

**Fig. 3:**
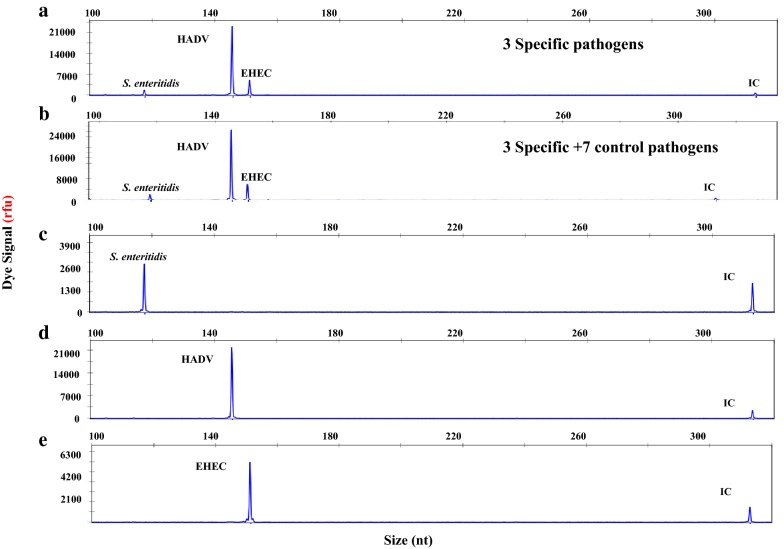
DP-HMGS robustly detected multiple DP signatures without interference between multiple signatures. **a** The plasmids of *S. enteritidis,* HADV and EHEC combined at different proportions (1 × 10^3^ copies, 1 × 10^5^ copies, and 1 × 10^4^ copies, respectively) produced specific peaks at 120 bp, 145 bp and 152 bp with low, high and midrange signal intensities (2600 rfu, 20,000 rfu and 6000 rfu, respectively). **b** The combined plasmids of three DPs with 7 negative control pathogen templates consistently showed the specific peaks of *S. enteritidis,* HADV and EHEC with no interference. Notably, the 313-bp peak for the IC was generated in each DP-HMGS reaction. The individually tested *S. enteritidis* (**c**), HADV (**d**), and EHEC (**e**) produced specific peaks at 120 bp, 145 bp, and 152 bp, respectively, with corresponding intensities of 2600 rfu, 20,000 rfu and 6000 rfu. Notably, the 313-bp peak for the IC was generated in each DP-HMGS reaction

DP-HMGS was designed specifically for application in the detection of polymicrobial infections. To demonstrate this capability, a mixture of 3 DP-associated plasmids—*S. enteritidis*, 1 × 10^3^ copies/μL; HADV, 1 × 10^5^ copies/μL; and EHEC, 1 × 10^4^ copies/μL—were combined and screened by the DP-HMGS assay. Three specific amplification peaks (*S. enteritidis*, 117.58 bp; HADV, 145.61 bp; EHEC, 151.19 bp) were observed, as shown in Fig. 3a. Again, the addition of the 7 negative control microbial species did not interfere with the generation of the specific *S. enteritidis*, HADV and EHEC peaks, and no FP signals were induced in the presence of these negative control microbes (Fig. 3b). One possible pitfall of multiplex assays is interference by a strong signal with the peaks of a weak signal, which could be expected in the case of polymicrobial infections with a highly abundant DP accompanied by less abundant DPs. We assessed whether microbial DNA with high template quantities could interfere with the detection of low-abundance DNA signatures. The results indicated that the signal magnitude generated in the DP-HMGS assay was identical whether all 3 DNA templates were tested in combination or individually (Fig. 3c–e). Thus, DP-HMGS robustly detected multiple DP signatures without interference by the signal for the dominant DNA template with those of low-abundance DNA templates.

### DP-HMGS can directly detect specific microbial signatures in faecal specimens

Before large-scale validation studies were conducted, we sought to determine whether DP-HMGS would perform “as expected” with healthy and diarrhoeal faecal specimens. A faecal specimen with confirmed *C. difficile* infection was compared against faecal specimens from healthy volunteers. The samples were simultaneously subjected to full genetic material extraction and a full cycle of DP-HMGS screening. The infected faecal specimens processed by DP-HMGS successfully exhibited the specific amplification signals: the target DP signal (*C. difficile*) and signals for all three quality control genes, namely, *B2* *M*, *RNaseP*, and IC (Additional file 5: Figure S3A). The non-diarrhoeal faecal specimens did not produce any signals except for those corresponding to the 3 quality control products (Additional file 5: Figure S3B). The concurrently run negative control reaction (ddH_2_O) did not produce any peaks, except for the expected peak for IC (Additional file 5: Figure S3C). These tests were repeated with consistent results using faecal specimens with other confirmed DP infections (data not shown). Thus, the results of DP-HMGS analysis of faecal specimens were similar to those obtained under the optimized conditions using microbial DNA and plasmids, motivating us to perform further studies to validate DP-HMGS performance in clinical settings.

### The clinical study demonstrated the uniformly high sensitivity and specificity of DP-HMGS in the detection of DPs in faecal specimens, in contrast to the non-uniform sensitivity of conventional methods

**Fig. 4:**
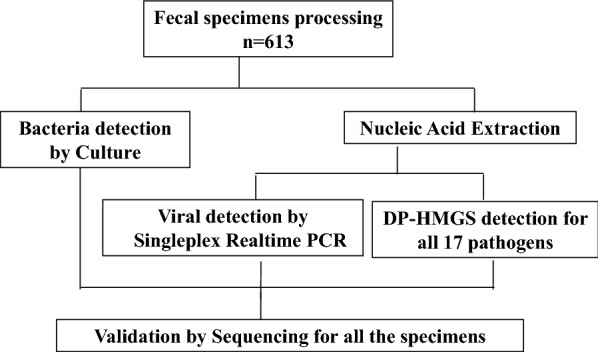
Workflow for faecal specimen processing. A schematic workflow chart depicting the processing of 613 faecal specimens for analysis, comparing conventional methods and DP-HMGS

To test the performance of DP-HMGS in clinical applications, we conducted a clinical study by analysing faecal specimens of 30 healthy volunteers and 613 hospital patients with suspected infectious diarrhoea. Among these patients, 319 (52.0%) were males and 294 (48.0%) were females. Loose stool was the most common symptomatic characteristic (37.4%), followed by watery stool (34.3%), mushy stool (22.0%), mucosal stool (5.4%) and bloody stool (0.9%). All of the faecal specimens collected from the recruited patients were assayed and processed as outlined in Fig. 4. For all the faecal specimens, the culture-based method was used to identify possible bacterial pathogens. Nucleic acids were extracted from the faecal specimens to perform singleplex real-time PCR for virus detection and DP-HMGS analysis. For bacterial identification, DP-HMGS was compared with the culture-based method. For virus identification, DP-HMGS was compared with singleplex real-time PCR. The specimens with inconsistent results between DP-HMGS and the culture-based method were further verified by conventional PCR and Sanger sequencing, which is considered the “gold standard” for gene detection [25, 26]. The primer sequences for conventional PCR and product sizes for Sanger sequencing are shown in Additional file 2: Table S2. Finally, the extracted nucleic acids were used for DNA sequencing as the most stringent reference to calculate sensitivities, specificities, and the predictive values of DP-HMGS, the conventional culture-based methods (for bacteria), and singleplex real-time PCR (for viruses).

DP-HMGS testing of faecal specimens from healthy individuals produced only the specific internal control peaks of Hum_RNA, Hum_DNA and IC, without any DP-specific peaks (data not shown). There were 345 positive DP specimens among the 613 faecal specimens validated by DNA sequencing, and 494 DPs were detected from these 345 positive specimens. The results consistently showed that the sensitivity and NPV of DP-HMGS were higher than those of the culture-based method for all bacteria (Tables 1 and 2). For virus detection, the sensitivity of DP-HMGS was as high as that of singleplex real-time PCR. The specificity and PPV of DP-HMGS were lower than those of the culture-based method for bacteria but were similar to those of singleplex real-time PCR for viruses (Tables 1 and 2). Thus, the efficacies of culture-based methods for bacterial pathogens were highly variable compared to the relatively uniform and sensitive performance of DP-HMGS. The single PCR assays conducted for the detection of a single viral pathogen were comparable with DP-HMGS.

**Table 1:** Comparison of conventional methods and Sanger sequencing for the detection of individual DPs

Bacteria	Culture	Sequencing		Sensitivity	Specificity	PPV	NPV	Accuracy
		+	−					
*Vibrio*	+	23	0	0.575	1.000	1.000	0.971	0.972
	−	17	573					
*S. typhimurium*	+	17	0	0.415	1.000	1.000	0.960	0.961
	−	24	572					
*S. enteritis*	+	2	0	0.333	1.000	1.000	0.993	0.993
	−	4	607					
*Shigella*	+	3	0	0.333	1.000	1.000	0.990	0.990
	−	6	604					
*C. difficile*	+	6	0	0.214	1.000	1.000	0.964	0.964
	−	22	585					
*C. jejuni*	+	12	0	0.267	1.000	1.000	0.946	0.947
	−	33	578					
*Y. enterocolitica*	+	0	0	0.000	1.000	–	0.998	0.998
	−	1	612					
EPEC	+	49	0	0.325	1.000	1.000	0.819	0.834
	−	102	462					
ETEC	+	11	0	0.379	1.000	1.000	0.970	0.971
	−	18	584					
EAEC	+	8	0	0.296	1.000	1.000	0.969	0.969
	−	19	586					
EIEC	+	2	0	0.250	1.000	1.000	0.990	0.990
	−	6	605					
EHEC	+	0	0	0.000	1.000	–	0.993	0.993
	−	4	609					
*E. coli* O157	+	1	0	0.167	1.000	1.000	0.992	0.992
	−	5	607					
Viruses	Singleplex real-time PCR							
Norovirus	+	31	2	1.000	0.997	0.939	1.000	0.997
	−	0	580					
Rotavirus	+	55	3	1.000	0.995	0.948	1.000	0.995
	−	0	555					
Adenovirus	+	8	1	1.000	0.998	0.888	1.000	0.995
	−	0	604					
Astrovirus	+	4	0	1.000	1.000	1.000	1.000	0.997
	−	0	609					

Outcomes of conventional detection methods: the results of culture-based methods for bacteria and singleplex real-time PCR for viruses were compared to those of Sanger sequencing. Sensitivity = TP/(TP + FN) × 100, Specificity = TN/(TN + FP) × 100; PPV and NPV were calculated as follows: PPV = TP/P, NPV = TN/N. Notably, the sensitivity of the culture-based method was very low and highly variable for various species, while the outcomes of PCR-based detection of viruses were consistent with the sequencing method

*DP*-*HMGS* diarrheal pathogens high-throughput multiple genetic detection system, *PPV* positive predict value, *NPV* negative predict value

**Table 2:** DP-HMGS exhibited uniformly high sensitivity of DP detection compared to the sequencing method

Pathogens	HMGS	Sequencing		Sensitivity	Specificity	PPV	NPV	Accuracy
		+	−					
*Vibrio*	+	39	1	0.975	0.998	0.975	0.998	0.997
	−	1	572					
*S. typhimurium*	+	41	1	1.000	0.998	0.976	1.000	0.998
	−	0	571					
*S. enteritis*	+	4	2	0.667	0.997	0.667	0.997	0.993
	−	2	605					
*Shigella*	+	9	1	1.000	0.998	0.900	1.000	0.998
	−	0	603					
*C. difficile*	+	28	0	1.000	1.000	1.000	1.000	1.000
	−	0	585					
*C. jejuni*	+	44	0	0.978	1.000	1.000	0.998	0.998
	−	1	568					
*Y. enterocolitica*	+	1	0	1.000	1.000	1.000	1.000	1.000
	−	0	612					
EPEC	+	151	0	1.000	1.000	1.000	1.000	1.000
	−	0	462					
ETEC	+	29	2	1.000	0.997	0.935	1.000	0.997
	−	0	582					
EAEC	+	27	0	1.000	1.000	1.000	1.000	1.000
	−	0	586					
EIEC	+	8	1	1.000	0.998	0.889	1.000	0.998
	−	0	604					
EHEC	+	4	0	1.000	1.000	1.000	1.000	1.000
	−	0	609					
*E. coli* O157	+	6	2	1.000	0.997	0.750	1.000	0.997
	−	0	605					
Norovirus	+	31	7	1.000	0.988	0.816	1.000	0.982
	−	0	575					
Rotavirus	+	55	2	1.000	0.996	0.964	1.000	0.990
	−	0	556					
Adenovirus	+	9	3	1.000	0.995	0.750	1.000	0.993
	−	0	601					
Astrovirus	+	4	0	1.000	1.000	1.000	1.000	0.997
	−	0	609					

The outcomes of DP-HMGS and sequencing-based DP detection methods were compared. Sensitivity = TP/(TP + FN) × 100, Specificity = TN/(TN + FP) × 100; PPV and NPV were calculated as follows: PPV = TP/P, NPV = TN/N. Notably, the sensitivity of DP-HMGS is high and comparable to that of sequencing for virtually all DP species

*DP*-*HMGS* diarrheal pathogens high-throughput multiple genetic detection system, *PPV* positive predict value, *NPV* negative predict value

### DP-HMGS corrects inaccurate conclusions regarding major causes of infectious diarrhoeas by summarizing the combined outcomes of conventional detection methods of major DPs

**Fig. 5:**
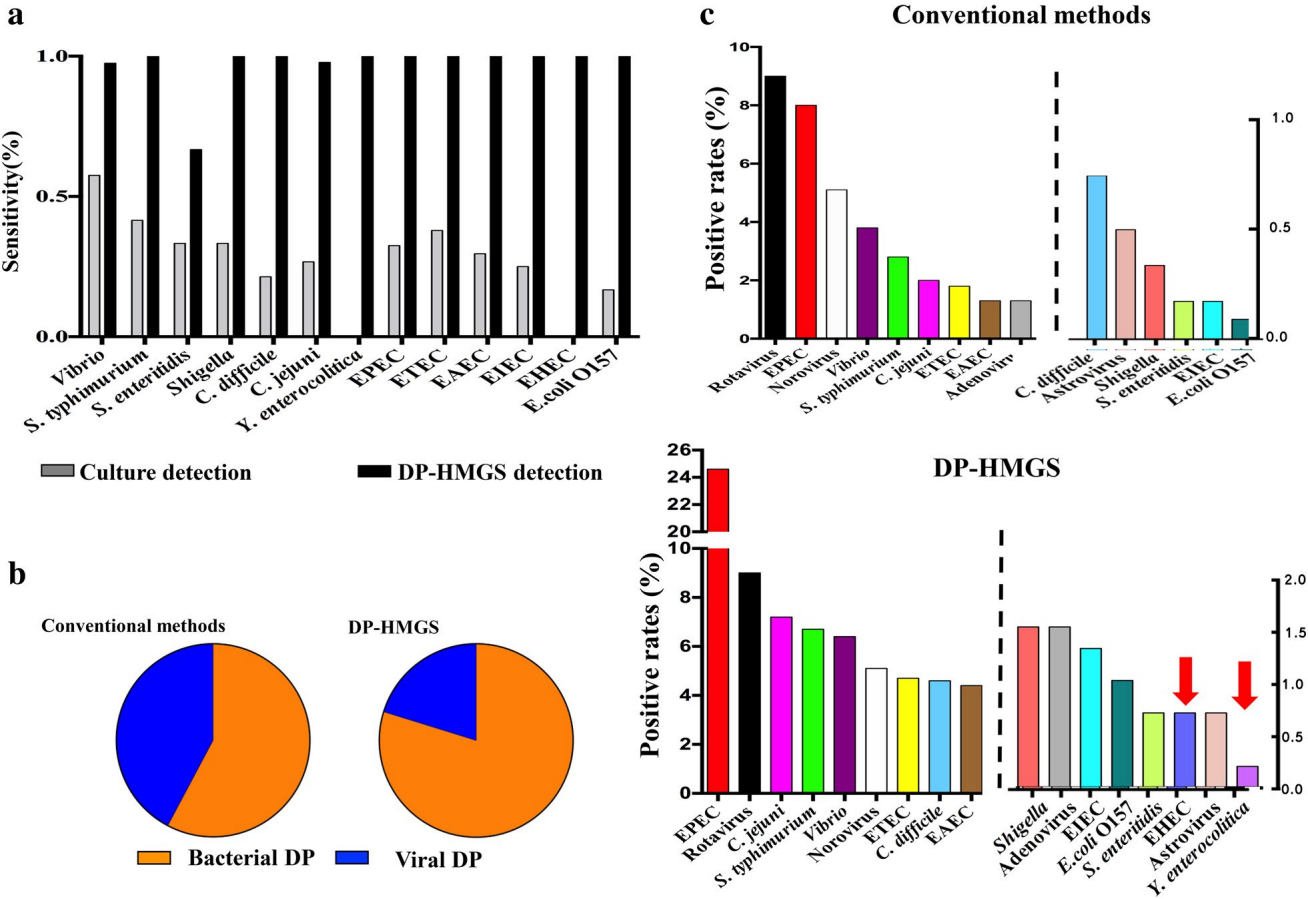
The uniformly high sensitivity of DP-HMGS corrected previous conclusions regarding the major causes of infectious diarrhoeas. **a** Comparisons of culture-based methods versus DP-HMGS with regard to sequencing-based detection of 13 diarrhoeal bacterial pathogens were conducted. The sensitivity of DP-HMGS for the detection of *Vibrio* (*P *= 0.000), *S. typhimurium* (*P *= 0.000), *Shigella* (*P *= 0.005), *C. difficile* (*P *= 0.000), *C. jejuni* (*P *= 0.000), EPEC (*P *= 0.000), ETEC (*P *= 0.000), EIEC (*P *= 0.003) and *E. coli* O157 (*P *= 0.008) was significantly lower than that of culture-based detection. Notably, the sensitivity of DP-HMGS was at least twice that of culture-based methods for most bacterial species and as virtually identical to that of sequencing. **b** Uniform sensitivity of DP-HMGS in detecting both bacterial and viral DPs corrected the ratios of bacterial versus viral causes of diarrhoeas, demonstrating the overwhelming importance of bacterial infections. **c** The epidemiological contribution of individual DP species was ranked based on the frequency of positive detection rates observed using conventional methods (top) and DP-HMGS (bottom). Notably, DP-HMGS corrected the epidemiological outcomes attained by combining the results of conventional methods, which were profoundly skewed by the low and highly variable sensitivity of culture-based methods in detecting bacterial DPs

High disparity among the detection parameters of various DPs using conventional detection methods (Table 1) prompted a careful analysis of the detection frequencies of the individual microbial species. Comparison of the detection rates by the culture-based method and DP-HMGS for 13 bacterial pathogens demonstrated that the sensitivity of DP-HMGS for all diarrhoeal bacteria was substantially higher than that of the culture-based method (Fig. 5a). The sensitivity of culture-based detection of different bacterial species was rather low (from 0 to 57.5%). Specifically, *E. coli* O157 (16.7%), *C. difficile* (21.4%), and EIEC (25%) exhibited positive detection rates, while EHEC and *Y. enterocolitica* were not detected at all. This sensitivity was in contrast to the comparable sensitivity of the detection of viral infection, for which individual PCR is a standard detection method. Because bacterial culture-based detection of bacterial DPs and PCR-based detection of viral DPs continue to be widely used to assess DP incidence in clinical studies [20, 27], we suspected that the marked disparity in the detection rates of these methods would substantially alter the conclusions regarding the epidemiological impact of each pathogen. We found that the significant disparity in the detection rates of viral versus bacterial pathogens and the highly variable sensitivities of culture-based detection for individual bacterial species distorted the conclusion regarding the relative importance of each pathogenic group (Fig. 5b) and individual DP species (Fig. 5c). Furthermore, the use of DP-HMGS resulted in complete remapping of the aetiological profile of the studied population with infectious diarrhoeas (Fig. 5c), with frequencies very similar to those obtained by sequencing (not shown). In addition to detecting marked changes in the distribution of DPs, DP-HMGS detected EHEC and *Y. enterocolitica*, which were completely missed by the culture-based detection approach. Collectively, these data show that the uniformly high sensitivity of detection achieved by DP-HMGS allowed the correction of epidemiological conclusions regarding major causes of infectious diarrhoeas, highlighting the prospect of using DP-HMGS as a tool for future clinical studies.

### The DP frequency distribution exhibited significant age variation

**Fig. 6:**
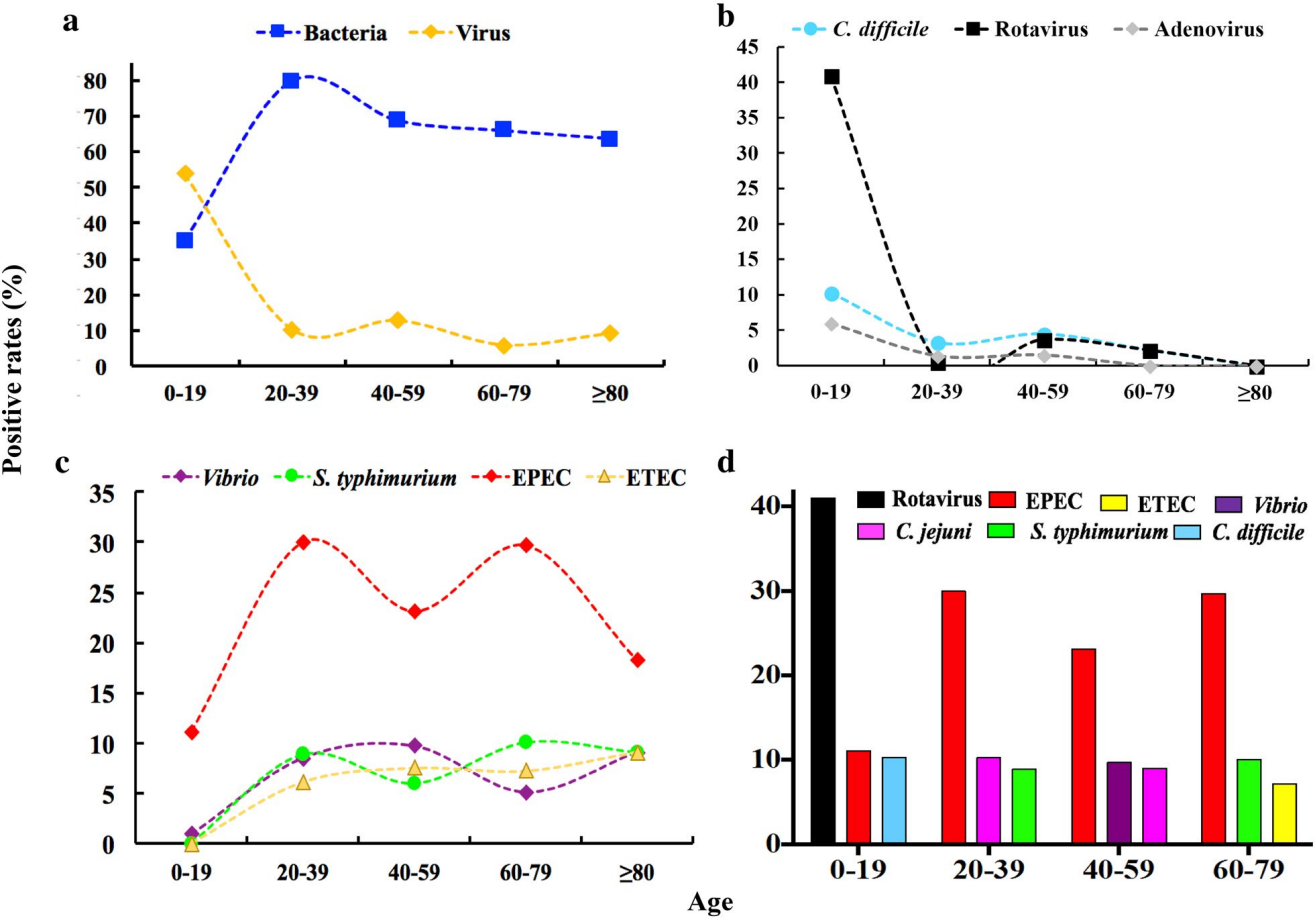
The DP frequency distribution exhibited significant age variation. **a** The positive detection rate and the trends in the distribution of total bacteria and total viruses. **b** The positive detection rate and the trends in the distribution of *C. difficile*, rotavirus and adenovirus. **c** The positive detection rate and the trends in the distribution of *Vibrio, S. typhimurium,* EPEC and ETEC. **d** The frequency of the predominant DPs in different age groups. Note that the P-values of the DPs selected in **b** were all less than 0.05. The Y-axis represents the positive DP detection rates, while the X-axis represents the different age groups

We expanded the analysis of the epidemiological distribution of DP frequencies in our patient cohort, analysing the incidence of DP infections in age- and gender-grouped subpopulations by DP-HMGS (Table 3). The epidemiological analysis demonstrated that the most common infectious bacterial pathogens were EPEC (24.6%), *C. jejuni* (7.2%)*, S. typhimurium* (6.9%) and *Vibrio* (6.5%). Rotavirus was the most common virus and was identified in 9.3% of the cases of DP infection, while other viruses, including norovirus (6.2%), adenovirus (2.0%), and astrovirus (0.7%), were responsible for a smaller portion of the infections. We observed significant differences in the distribution of DPs within age groups, and the results revealed that the occurrence of most of the infections was age dependent. The most dramatic, age-related differences were observed between total bacterial and viral infections; a major change in the infection profiles was observed between children and adults (Table 3, Fig. 6a). Viral pathogens were responsible for the largest number of detected cases in the youngest (≤ 19 years old) age group (53.9% cases, 63 out of 117), while bacteria accounted for only 35% of the cases (41 out of 117). This trend in the age distribution of viral infection was driven entirely by the dynamics of rotavirus and adenovirus infections (Fig. 6b), which together accounted for 87.3% (55 out of 63) of the viral infections in young patients and nearly 47.0% (55 out of 117) of all the specimens in this age group but were only sporadically detected or absent in the remaining age groups (Table 3). In addition to high incidences of rotavirus and adenovirus, we observed a significant peak for a single bacterial DP, namely, the pathogenic *C. difficile*, (Fig. 6b) in these paediatric patients. Further analysis revealed that most of the rotavirus (75.0%, 36/48), adenovirus (41.7%, 5/12) and pathogenic *C. difficile* (66.7%, 8/12) were detected in the 0–3-year-old age group. In contrast, bacterial infections predominated in all the adult age groups (20 years old and above), accounting for 63.6–79.8% of the cases, while viral infections accounted for approximately 5.8–12.7% of the cases at these age intervals (Fig. 6b, c). Predominant in all the adult age groups were 4 bacterial DPs, namely, *Vibrio*, *S. typhimurium*, EPEC, and ETEC (Fig. 6c, d). With the exception of EPEC, which was the most common in all the adult groups, the frequencies of the DPs varied in all the age groups, as exemplified by the “top 3” DPs for each age interval (Fig. 6d). Finally, the incidences of individual DP infections between the gender groups were similar for most pathogens; however, *Shigella* and EIEC, albeit relatively rare, exhibited a very strong predilection for occurring in female patients (Table 3).

**Table 3:** DP-HMGS analysis revealed significant effects of age and gender on the frequencies of common DP infections

Pathogens	Positive numbers (n[%]) (n = 613)	**Age (n[%])**					*P* value	Gender (n[%])		*P* value
		0–19 (n = 117)	20–39 (n = 213)	40–59 (n = 134)	60–79 (n = 138)	≥ 80 (n = 11)		Male (n = 319)	Female (n = 294)	
Bacteria	401 (65.4)	41 (35.0)	170 (79.8)	92 (68.7)	91 (65.9)	7 (63.6)	0.000	215 (67.4)	186 (63.3)	0.282
*Vibrio*	40 (6.5)	1 (0.9)	18 (8.5)	13 (9.7)	7 (5.1)	1 (9.1)	0.011	24 (7.5)	16 (5.4)	0.297
*S. typhimurium*	42 (6.9)	0 (0.0)	19 (8.9)	8 (6.0)	14 (10.1)	1 (9.1)	0.001	23 (7.2)	19 (6.5)	0.714
*S. enteritidis*	6 (1.0)	1 (0.9)	3 (1.4)	2 (1.5)	0 (0.0)	0 (0.0)	0.636	2 (0.6)	4 (1.4)	0.357
*Shigella*	10 (1.6)	1 (0.9)	4 (1.9)	3 (2.2)	1 (0.7)	1 (9.1)	0.266	1 (0.3)	9 (3.1)	0.009
*C. difficile*	28 (4.6)	12 (10.3)	7 (3.3)	6 (4.5)	3 (2.2)	0 (0.0)	0.042	17 (5.3)	11 (3.7)	0.347
*C. jejuni*	44 (7.2)	6 (5.1)	22 (10.3)	12 (9.0)	4 (2.9)	0 (0.0)	0.060	21 (6.6)	23 (7.8)	0.552
*Y. enterocolitica*	1 (0.2)	0 (0)	1 (0.5)	0 (0)	0 (0)	0 (0)	1.000	1 (0.3)	0 (0)	0.337
EPEC	151 (24.6)	13 (11.1)	64 (30.0)	31 (23.1)	41 (29.7)	2 (18.2)	0.020	85 (26.6)	66 (22.4)	0.228
ETEC	31 (5.1)	0 (0)	13 (6.1)	7 (7.5)	10 (7.2)	1 (9.1)	0.014	16 (5.0)	15 (5.1)	0.961
EAEC	27 (4.4)	3 (2.6)	10 (4.7)	6 (4.5)	8 (5.8)	0 (0.0)	0.794	17 (5.3)	10 (3.4)	0.245
EIEC	9 (1.5)	1 (0.9)	3 (1.4)	3 (2.2)	1 (0.7)	1 (9.1)	0.235	1 (0.3)	8 (2.7)	0.017
EHEC	4 (0.7)	2 (1.7)	2 (0.9)	0 (0)	0 (0)	0 (0)	0.311	3 (0.9)	1 (0.3)	0.625
*E. coli* O157	8 (1.3)	1 (0.9)	4 (1.9)	1 (0.7)	2 (1.4)	0 (0)	0.883	4 (1.3)	4 (1.4)	1.000
Viruses	111 (18.1)	63 (53.9)	22 (10.3)	17 (12.7)	8 (5.8)	1 (9.1)	0.000	56 (17.6)	55 (18.7)	0.711
Norovirus	38 (6.2)	6 (5.1)	17 (8.0)	9 (6.7)	5 (3.6)	1 (9.1)	0.426	20 (6.3)	18 (6.1)	0.940
Rotavirus	57 (9.3)	48 (41.0)	1 (0.5)	5 (3.7)	3 (2.2)	0 (0)	0.000	31 (9.7)	26 (8.8)	0.710
Adenovirus	12 (2.0)	7 (6.0)	3 (1.4)	2 (1.5)	0 (0)	0 (0)	0.019	3 (0.9)	9 (3.1)	0.079
Astrovirus	4 (0.7)	2 (1.7)	1 (0.5)	1 (0.7)	0 (0)	0 (0)	0.377	2 (0.6)	2 (0.7)	1.000
Total	512 (83.5)	104 (88.9)	192 (90.1)	109 (81.3)	99 (71.7)	8 (72.7)	0.000	271 (85.0)	241 (82.0)	0.320

The outcomes of positive DP-HMGS detection were categorized into appropriate age and gender groups. Notably, incidences of some DPs exhibited significant age and gender dependence

### The superior potential of DP-HMGS to detect polymicrobials reveals an unexpectedly high incidence of multifactorial diarrhoea

**Fig. 7:**
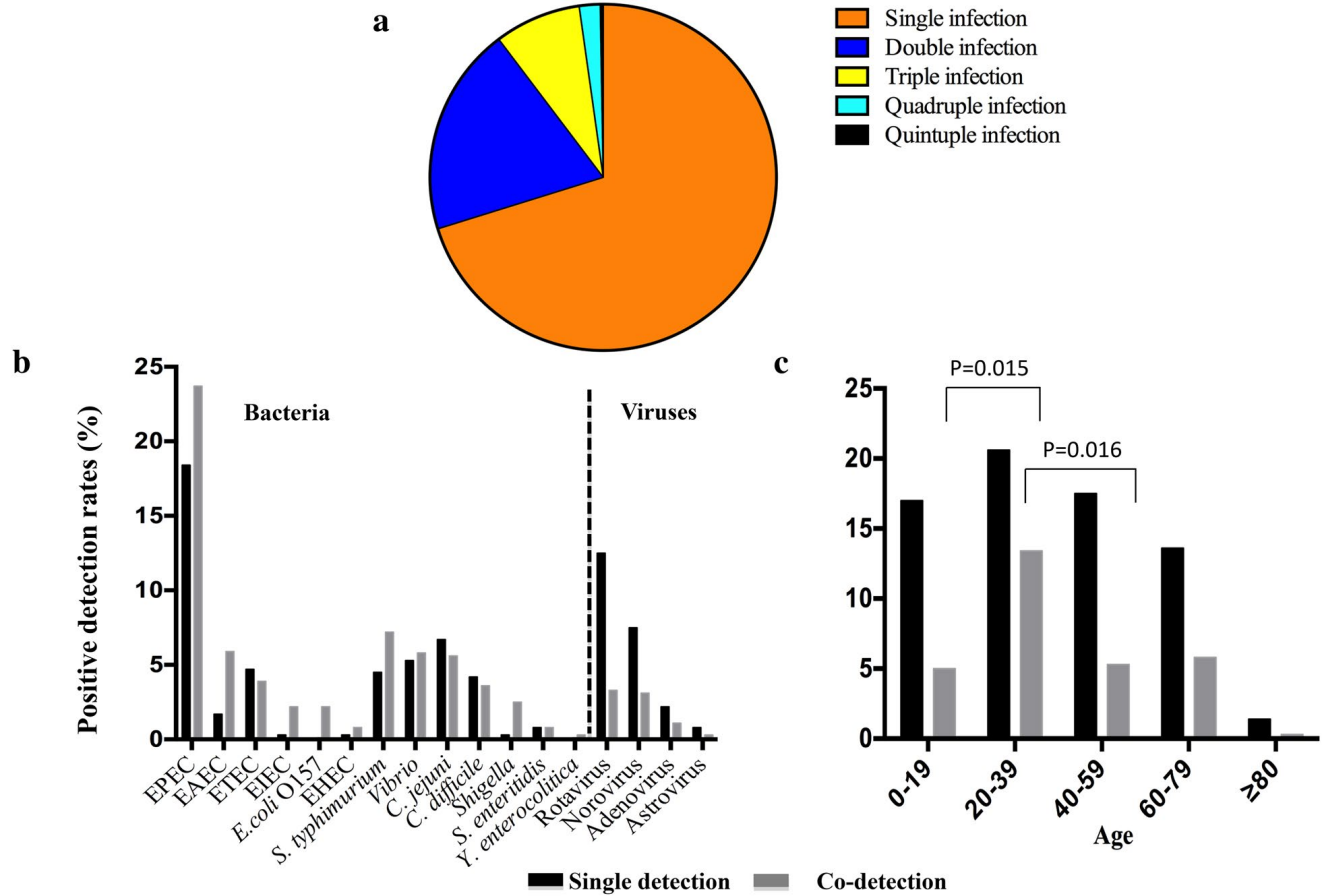
DP-HMGS application revealed an unexpectedly high incidence of multifactorial diarrhoea. **a** Infection profiles of 17 DPs; the different colours represent single infection, double infection, triple infection, quadruple infection and quintuple infection. **b** The positive detection rates of single and polymicrobial infections of 17 DPs are shown. **c** Analysis of the correlation between the positive detection rates of single and polymicrobial infections and age groups for 17 DPs. Darker and lighter colours in the histogram indicate single and co-pathogen detection, respectively

The subsequent evaluation of 613 faecal specimens for polymicrobial infections revealed that approximately one-third of the confirmed infectious diarrhoeas were polymicrobial, which was unexpected (Fig. 7a). Interestingly, some DPs tended to exhibit polymicrobial infections more often than they exhibited individual infections, such as EPEC, EAEC, EIEC, *E. coli* O157, EHEC, *S. typhimurium, Vibrio*, *Shigella* and *Y. enterocolitica*. As shown in Additional file 6: Table S3, the most commonly detected polymicrobial infections were EPEC and *Vibrio*, EPEC and EAEC, EPEC and *S. typhimurium*, and EPEC and *C. jejuni*. In addition, there were some triple bacterial infections, such as EPEC, ETEC and EAEC and EPEC, EAEC and *S. typhimurium*. In addition to the multi-bacterial infections, we also observed polymicrobial infections with bacteria and viruses, e.g., *C. difficile* and rotavirus; EPEC and rotavirus; and EPEC, norovirus and *Vibrio*. We found that among most of the bacterial DPs, the number of polymicrobial infections was as high as or greater than the number of single infections. In particular, for pathogenic *E. coli*, the number of polymicrobial infections was much greater than that of single infections. In contrast, the viral infections exhibited a high prevalence of single DPs and a low number of co-detected DPs, especially in rotavirus- and norovirus-infected patients (Fig. 7b). Finally, we observed that the frequency of polymicrobial infections was greatest among the 20–39-year-old age group (Fig. 7c), members of which were significantly more likely to develop polymicrobial infections than the members of the 0–19-year-old (*P *= 0.015) and 40–59-year-old patient groups (*P *= 0.016). Thus, we revealed unexpectedly high rates of polymicrobial infections in the studied patient population, particularly in patients in the 20–39-year-old age group.

## Discussion

Diarrhoeal infections represent a class of highly infectious diseases that rapidly spread and significantly impact the health of large populations, with the most severe cases leading to death [5]. Causes of gastroenteritis are predominantly infectious, triggered by multiple classes of bacterial, viral and parasitic pathogens or other non-infectious factors [27]. Detection of DPs in these cases is not routinely conducted or is conducted using conventional detection methods, such as culture-based methods and singleplex real-time PCR. These methods are relatively time consuming, costly (due to the need for the application of multiple approaches), and labour intensive and are usually limited to the detection of a single pathogen or a group of closely related pathogens per test [28, 29]. In this study, we established and optimized a rapid, sensitive, specific and well-controlled DP identification and screening assay—DP-HMGS—which allowed the detection of 19 classes of pathogenic DPs simultaneously in faecal specimens. Systematic analysis of 613 clinical specimens with this highly sensitive and specific DP-HMGS assay revealed that (1) the DP-HMGS method was more sensitive and could detect more pathogenic bacteria than the culture-based method while maintaining sensitivity levels comparable to those of single PCR-based detection; (2) several major aetiological agents remained frequently underdiagnosed as important causes of acute infectious diarrhoeas when assessed solely using conventional methods, leading to incorrect conclusions regarding major causes of infectious diarrhoeas; (3) the DP frequency distribution detected by DP-HMGS exhibited significant age variation; and (4) approximately 1/3 of the cases of infectious diarrhoeas were co-induced by multiple pathogens, with some DPs preferentially occurring as co-infecting agents.

Culture-based methods are the most conventional methods and continue to be commonly used for the detection of bacterial causes of diarrhoea as a “gold standard” for aetiological diagnosis in a vast majority of health centres. However, culture-based methods have several significant limitations that restrict the use of these methods as the “first line” of DP screening [28, 29]. These limitations include the requirement of up to several days of growth before analysis, requirement of variable media and culture conditions for various species, problems associated with overgrowth of non-pathogenic bacteria that are abundant in the gut, and limited sensitivity due to technical limitations of incubation environments. Finally, a number of strictly anaerobic but important bacterial species, such as *C. difficile* are difficult to be cultured under routine laboratory conditions due to the requirement of specific equipment [30, 31]. Detection of viruses is commonly conducted by singleplex real-time PCR. However, the use of individual PCRs to screen multiple pathogens is tedious and costly [29, 32, 33]. Our present study demonstrates that the detection levels for most bacterial pathogens using culture-based methods alone were unacceptably low. Indeed, for most pathogens, the detection levels were 30% or lower (Table 1). Furthermore, low and non-uniform culture detection rates resulted in a significant bias, with the epidemiological data showing a very high impact of viral infection and greatly underestimating that of bacteria (Fig. 5). DP-HMGS allowed us to overcome the significant disparity in the sensitivities of the conventional detection methods, bringing the accuracy of the epidemiological map of DPs to the level obtained by sequencing methods (Table 2, Fig. 5). The major advantages of the DP-HMGS platform that allowed its successful implementation were high sensitivity, specificity and accuracy for the identification of DP and, most importantly, a relatively uniform performance for all DPs. One potential drawback of DNA/RNA-based molecular detection methods (including DP-HMGS) is that these methods do not reveal whether an infectious agent is viable. However, considering the detection limit (10^2^–10^3^ copies/µL), DP-HMGS detected only significantly abundant microbes in the GI tract, in turn strongly suggesting the association of the microbes with the disease. Further studies are needed to definitively address whether the detection of microbes at this level always signifies the presence of viable microbes in the GI tract.

In terms of epidemiological findings, we showed that EPEC was the DP most frequently associated with infectious diarrhoeas in Shanghai, accounting for 24.6% of cases (Table 3); this finding was consistent with previous reports from China [34] and Singapore [35]. In contrast, a report by Moreno et al. concluded that EAEC rather than EPEC was the major cause of diarrhoea [36]. While its relative contributions to diarrhoea epidemiology have often been inconsistent between reports [37, 38], EPEC continues to be the most prevalent type of pathogenic *E. coli* found in industrialized countries [39, 40]. A strong association between EPEC and diarrhoea in children has been reported [39], and yet, we also found a relatively high prevalence of EPEC in older patients in our study (Fig. 6c). In the study of the age distribution of pathogens, the predominant DP frequencies varied among all age groups, as exemplified by the “top 3” DPs in each age interval (Fig. 6d), which might be attributed to the different lifestyles, food preferences and immune statuses of the patients in different age groups. However, these disparities were likely influenced by different research periods and the variety of methods applied for detection. Rotavirus was most frequently found and highly prevalent among young patients (0–19 ages) but less prevalent in other age groups, which was consistent with the results of most epidemiological studies [17, 41]. Another infection that predominantly impacted patients ≤ 19 years old was pathogenic *C. difficile*. This result is consistent with a report by Buss et al. that showed a high proportion of *C. difficile* in the infectious DPs detected from paediatric faecal specimens using the FilmArray gastrointestinal panel [42]. The clinical practice guidelines for *C. difficile* infection in adults and children do not recommend testing for *C. difficile* in children less than 2 years old unless other causes of disease have been explicitly excluded [43]. In our study, we specifically detected pathogenic *C. difficile* in 0–2-year-old patients with diarrhoea symptoms without a confirmed clinical diagnosis of other causes of disease. These outcomes provided useful diagnostic clues, suggesting that in most of these cases, the major cause of diarrhoea was pathogenic *C. difficile*. Thus, future studies are needed to establish whether the detection of pathogenic *C. difficile* in young children in these circumstances is important for clinical diagnosis and treatment.

The final important finding in our study was a high frequency of polymicrobial infection with two or more DPs, relative to previously reported data [44, 45], which demonstrated another level of complexity in determining the aetiology of diarrhoea, which could be offset by the use of multiplex detection systems such as DP-HMGS. Because of the limitations of conventional methods, polymicrobial infections usually go undetected, and none of the polymicrobial infections could be identified by culture-based methods in our study [46]. The polymicrobial infections identified by DP-HMGS accounted for 1/3 of the positive specimens detected (Fig. 7a), showing that the *E. coli* subgroups were much more common in polymicrobial infections than as the cause of single DP infections (Fig. 7b). This result may be due to the *E. coli* pathogroup requiring partner pathogens to cause severe diarrhoeal disease [46–48]. Among the polymicrobial infections, we found that EPEC and *Vibrio* presented the highest ratio (Additional file 6: Table S3). One possible reason for this observation could be that the isolation rate of EPEC was the highest (24.6%), and *Vibrio* was the most frequently isolated pathogen from seawater and seafood, and the consumption of contaminated seafood appears to be one of the major causes of acute diarrhoea in Shanghai [49]. Finally, we noticed that the proportion of polymicrobial infections in the 20–39-year-old age group was significantly higher than that in the 40–59-year-old age group (Fig. 7c), which may be attributed to the different lifestyles of younger adults, including more frequent social activity, travelling and moving than the older group.

However, these findings regarding polymicrobial infections could be a result of colonization by another DP because differentiation between colonization and true polymicrobial infections is relatively difficult (regardless of the detection methods) [50]. One advantage of DP-HMGS was that specific pathogenic gene sequences were used for the detection of *C. difficile, S. typhimurium, Shigella* and *E. coli* pathogenic strains (EPEC, ETEC, EAEC, EIEC, EHEC and *E. coli* O157). Thus, DP-HMGS could be used to distinguish between colonizing and pathogenic bacteria. Nevertheless, for the remaining DPs, which did not have specific pathogenicity-associated genes, colonization could not be distinguished from an active pathogenic process.

## Conclusions

In summary, we developed and optimized a rapid, sensitive, highly specific multiplex DP identification system, namely, DP-HMGS, which allowed the detection of 19 classes of viral and bacterial DPs via a single test. Systematic analysis of 613 clinical specimens with the DP-HMGS assay significantly remapped the aetiology of infectious diarrhoeas in Shanghai. This result included the detection of underdiagnosed or previously undetected bacterial pathogens and unexpectedly high levels of infections co-induced by multiple DPs. Our study documents that DP-HMGS provides a highly effective and time-saving alternative for the clinical diagnosis of faecal specimens. We propose that the widespread use of such assays on a global scale would be helpful to update and correct the epidemiological map of major causes of diarrhoeal infections and promote rational treatments of infectious diarrhoeas.

## Additional files

**Additional file 1: Table S1.** Species-specific genes, primer sequences and product sizes for DPs in DP-HMGS. The designed primer sets, species-specific genes and the corresponding amplicon sizes for the molecular detection of 6 classes of viral and 13 classes of bacterial DPs as well as 3 quality controls for the DP-HMGS assay.

**Additional file 2: Table S2.** Conventional PCR primer sequences and product sizes for Sanger sequencing. The designed primer sets and the corresponding amplicon sizes for molecular detection of 6 classes of viral and 13 classes of bacterial DPs as well as 3 quality controls for Sanger sequencing.

**Additional file 3: Figure S1.** Primers designed for DP-HMGS effectively detect DPs in individual PCRs. In the DP-HMGS assay reaction system with all DP signature primers present, the nucleic acid templates for each of the pathogens were tested individually. Each class of diarrhoeal pathogens was successfully detected by DP-HMGS. The specific peaks individually appeared at 209 bp for *C. jejuni* (A), 136 bp for *Shigella* (B), 202 bp for *C. difficile* (C), 159 bp for HASV (D), 226 bp for norovirus (E), 190 bp for ETEC (F), 152 bp for EHEC (G), 251 bp for EPEC (H), 292 bp for EAEC (I), 129 bp for EIEC (J), 308 bp for rotavirus (K), 120 bp for *S. enteritidis* (L), 113 bp for *S. typhimurium* (M), 196 bp for *Vibrio* (N), 145 bp for HADV (O), 165 bp for *Y. enterocolitica* (P) and 218 bp for *E. coli* O157 (Q). Notably, the peaks in A-Q at 313 bp were for the IC, and all gene targets were specifically amplified without non-specific amplification by individual PCR assays.

**Additional file 4: Figure S2.** The DP-HMGS assay was highly specific for DP identification. (A) In the positive control, DP-HMGS detection of DP was conducted by simultaneous detection of the plasmid templates shown here for 17 pathogens and 3 quality controls. All the targets (from left to right: hum_RNA, *S. typhimurium, S. enteritidis,* EIEC*, Shigella,* HADV, EHEC, HASV, *Y. enterocolitica,* ETEC, *Vibrio, C. difficile, C. jejuni, E. coli* O157, norovirus, hum_DNA, EPEC, EAEC, rotavirus, IC) could be specifically detected by DP-HMGS. (B–H) DP-HMGS assay results obtained using 7 individual plasmid templates from *H. pylori, E. coli* DH5α*, P. aeruginosa, S. aureus, P. shigelloides,* sapovirus, and non-pathogenic *E. coli* showed no pathogen-specific peak. (I) DP-HMGS assay results obtained by using ddH_2_O as a DNA template showed no pathogen-specific peak. DP-specific genes produced signals only in the positive controls, not in the negative control, which consisted of pathogens that could potentially interfere with specific pathogen detection. Notably, the peaks in B-H at 313 bp were for the IC.

**Additional file 5: Figure S3.** DP-HMGS could directly detect specific microbial signatures in faecal specimens. (A) The human faecal specimen with a specific DP infection produced a *C. difficile*-specific peak at 202 bp, as well as specific peaks for the human internal RNA control, human internal DNA control and internal positive test control at 106 bp, 233 bp and 313 bp, respectively. (B) The faecal specimens from non-infectious diarrhoea patients produced only the specific peaks of Hum_RNA, Hum_DNA and IC at 106 bp, 233 bp and 313 bp, respectively. (C) ddH_2_O showed only the specific peak for the IC at 313 bp.

**Additional file 6: Table S3.** Frequency of the combinations of polymicrobial infections. The combinations and the percentages of the most common polymicrobial DP infections include double infections, triple infections, multi-bacterial infections and bacterial/viral polymicrobial infections.

## Abbreviations

DP: diarrhoeal pathogens
DP-HMGS: high-throughput multiplex genetic detection system for diarrhoeal pathogen detection
HASV: human astrovirus
NorV: norovirus II
HADV: human adenovirus
RoVA: rotavirus A
RoVB: rotavirus B
RoVC: rotavirus C
*C. jejuni*: *Campylobacter jejuni*
*C. difficile*: *Clostridium difficile*
*S. enteritidis*: *Salmonella enteritidis*
*S. typhimurium*: *Salmonella typhimurium*
*Y. enterocolitica*: *Yersinia enterocolitica*
*H. pylori*: *Helicobacter pylori*
*S. aureus*: *Staphylococcus aureus*
ETEC: enterotoxigenic *E. coli*
EHEC: enterohemorrhagic *E. coli*
EPEC: enteropathogenic *E. coli*
EAEC: enteroaggregative *E. coli*
EIEC: enteroinvasive *E. coli*
