# Supplementary material for: Clinical application of a multiplex genetic pathogen detection system remaps the aetiology of diarrhoeal infections in Shanghai
Source: Gut Pathog. 2018 Sep 11;10:37. doi: 10.1186/s13099-018-0264-7 (PMC6134694; doi:10.1186/s13099-018-0264-7)
Supplement: Supplementary file 1 — Additional file 1: Table S1. Species-specific genes, primer sequences and product sizes for DPs in DP-HMGS. The designed primer sets, species-specific genes and the corresponding amplicon sizes for the molecular detection of 6 classes of viral and 13 classes of bacterial DPs as well as 3 quality controls for the DP-HMGS assay. [file 13099_2018_264_MOESM1_ESM.ppt]

## Slide 1
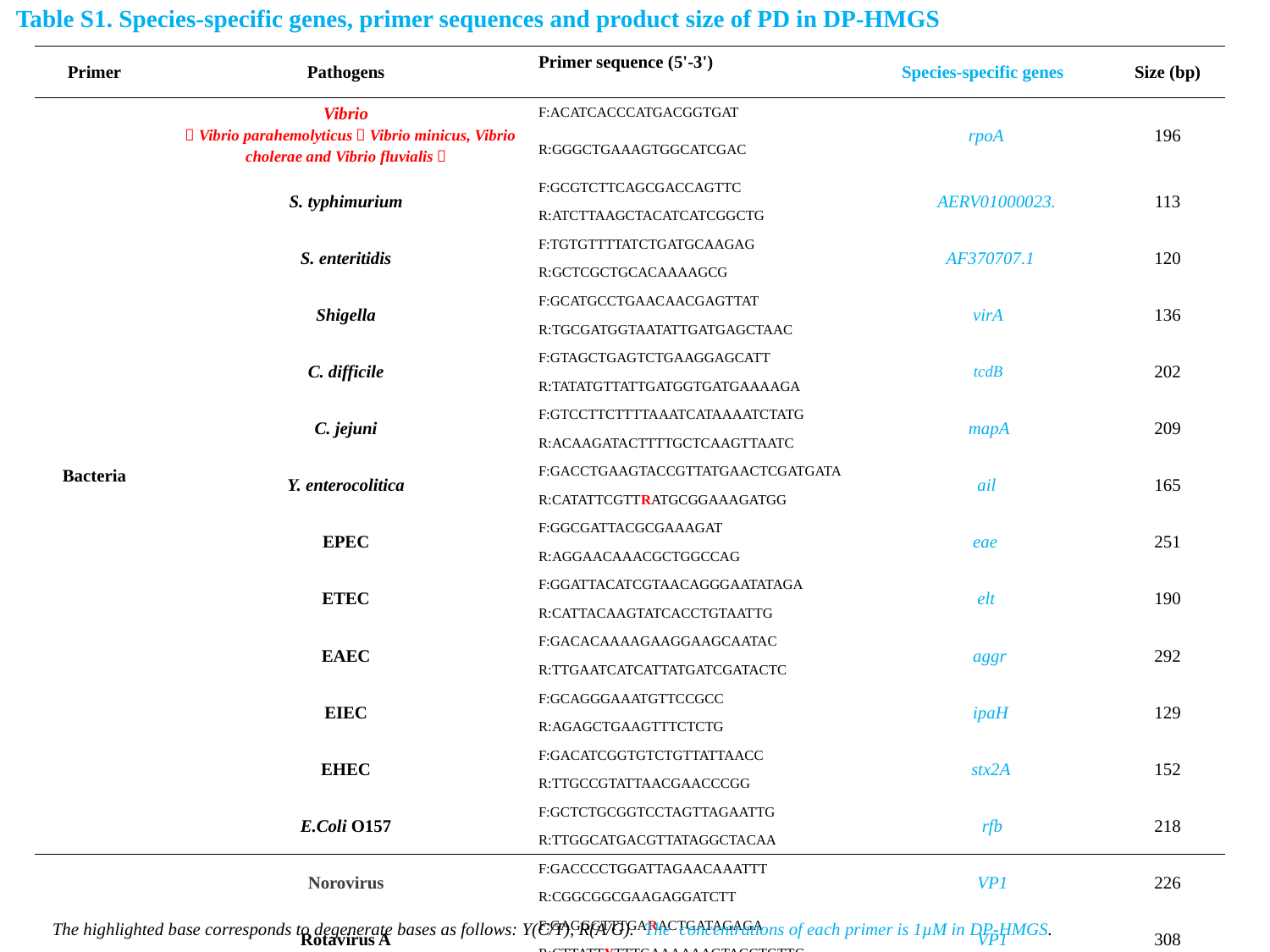

Table S1. Species-specific genes, primer sequences and product size of PD in DP-HMGS
| Primer | Pathogens | Primer sequence (5'-3') | Species-specific genes | Size (bp) |
| --- | --- | --- | --- | --- |
| Bacteria | Vibrio （Vibrio parahemolyticus，Vibrio minicus, Vibrio cholerae and Vibrio fluvialis） | F:ACATCACCCATGACGGTGAT | rpoA | 196 |
| | | R:GGGCTGAAAGTGGCATCGAC | | |
| | S. typhimurium | F:GCGTCTTCAGCGACCAGTTC | AERV01000023. | 113 |
| | | R:ATCTTAAGCTACATCATCGGCTG | | |
| | S. enteritidis | F:TGTGTTTTATCTGATGCAAGAG | AF370707.1 | 120 |
| | | R:GCTCGCTGCACAAAAGCG | | |
| | Shigella | F:GCATGCCTGAACAACGAGTTAT | virA | 136 |
| | | R:TGCGATGGTAATATTGATGAGCTAAC | | |
| | C. difficile | F:GTAGCTGAGTCTGAAGGAGCATT | tcdB | 202 |
| | | R:TATATGTTATTGATGGTGATGAAAAGA | | |
| | C. jejuni | F:GTCCTTCTTTTAAATCATAAAATCTATG | mapA | 209 |
| | | R:ACAAGATACTTTTGCTCAAGTTAATC | | |
| | Y. enterocolitica | F:GACCTGAAGTACCGTTATGAACTCGATGATA | ail | 165 |
| | | R:CATATTCGTTRATGCGGAAAGATGG | | |
| | EPEC | F:GGCGATTACGCGAAAGAT | eae | 251 |
| | | R:AGGAACAAACGCTGGCCAG | | |
| | ETEC | F:GGATTACATCGTAACAGGGAATATAGA | elt | 190 |
| | | R:CATTACAAGTATCACCTGTAATTG | | |
| | EAEC | F:GACACAAAAGAAGGAAGCAATAC | aggr | 292 |
| | | R:TTGAATCATCATTATGATCGATACTC | | |
| | EIEC | F:GCAGGGAAATGTTCCGCC | ipaH | 129 |
| | | R:AGAGCTGAAGTTTCTCTG | | |
| | EHEC | F:GACATCGGTGTCTGTTATTAACC | stx2A | 152 |
| | | R:TTGCCGTATTAACGAACCCGG | | |
| | E.Coli O157 | F:GCTCTGCGGTCCTAGTTAGAATTG | rfb | 218 |
| | | R:TTGGCATGACGTTATAGGCTACAA | | |
| Viruses | Norovirus | F:GACCCCTGGATTAGAACAAATTT | VP1 | 226 |
| | | R:CGGCGGCGAAGAGGATCTT | | |
| | Rotavirus A | F:GAGGGTTTGARACTGATAGAGA | VP1 | 308 |
| | | R:CTTATTYTTTGAAAAAAGTAGCTGTTC | | |
| | Rotavirus B | F:CTCTCACATATGGAGTATTAGCTGA | VP1 | 308 |
| | | R:GGATGCAACRCCATGATATCTTATTATTT | | |
| | Rotavirus C | F:TGTCAAGCTATGATGTTCACAATTTCT | VP1 | 308 |
| | | R:GGTGATGAYACTAATTTYGCTAATGAT | | |
| | Adenovirus | F:CAACATCGGCACCCCTCT | fiber | 145 |
| | | R:GGACCAAGATTGATTATTAACTTAGAG | | |
| | Astrovirus | F:TAGRAGACAGCCCGGAC | capsid protein | 159 |
| | | R:GTGCCRCTGGTGTTTGA | | |
| Controls | Hum\_RNA | F: GATGAGTATGCCTGCCGTG | B2M | 106 |
| | | R: ATGCGGCATCTTCAAACCT | | |
| | Hum\_DNA | F: GTGGATGCTACTTGTCCAATGATG | RNaseP | 233 |
| | | R: ACAATTCTCCGATCCGTCCCTAAC | | |
| | IC | F: GTGGCCGCTTTTCTGGATTCAT | modified Kanr | 313 |
| | | R: TGAAGGCACAGTCGAGGCTG | | |
The highlighted base corresponds to degenerate bases as follows: Y(C/T), R(A/G). The concentrations of each primer is 1µM in DP-HMGS.
