# Supplementary material for: Clinical application of a multiplex genetic pathogen detection system remaps the aetiology of diarrhoeal infections in Shanghai
Source: Gut Pathog. 2018 Sep 11;10:37. doi: 10.1186/s13099-018-0264-7 (PMC6134694; doi:10.1186/s13099-018-0264-7)
Supplement: Supplementary file 2 — Additional file 2: Table S2. Conventional PCR primer sequences and product sizes for Sanger sequencing. The designed primer sets and the corresponding amplicon sizes for molecular detection of 6 classes of viral and 13 classes of bacterial DPs as well as 3 quality controls for Sanger sequencing. [file 13099_2018_264_MOESM2_ESM.ppt]

## Slide 1
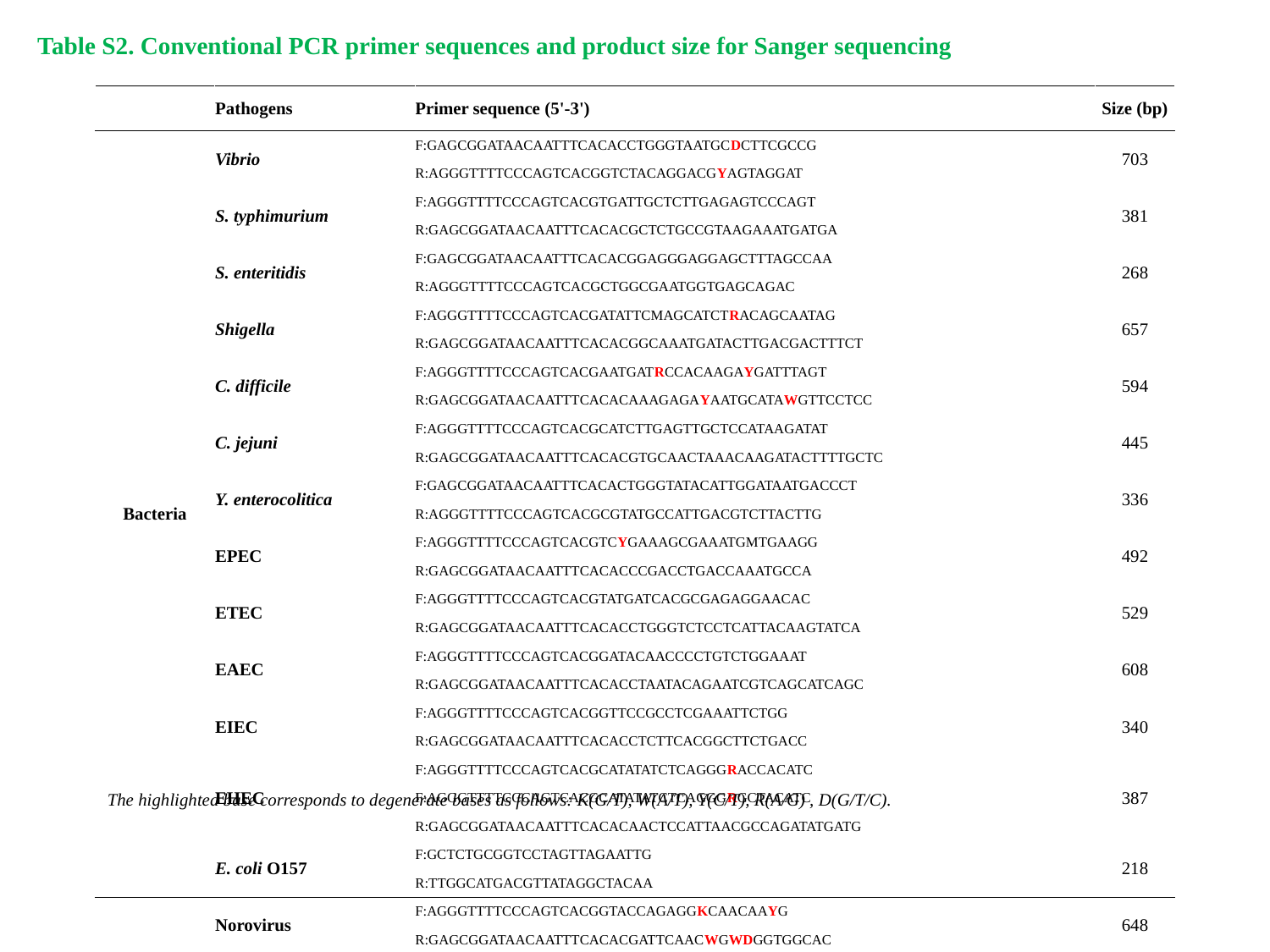

Table S2. Conventional PCR primer sequences and product size for Sanger sequencing
| | Pathogens | Primer sequence (5'-3') | Size (bp) |
| --- | --- | --- | --- |
| Bacteria | Vibrio | F:GAGCGGATAACAATTTCACACCTGGGTAATGCDCTTCGCCG | 703 |
| | | R:AGGGTTTTCCCAGTCACGGTCTACAGGACGYAGTAGGAT | |
| | S. typhimurium | F:AGGGTTTTCCCAGTCACGTGATTGCTCTTGAGAGTCCCAGT | 381 |
| | | R:GAGCGGATAACAATTTCACACGCTCTGCCGTAAGAAATGATGA | |
| | S. enteritidis | F:GAGCGGATAACAATTTCACACGGAGGGAGGAGCTTTAGCCAA | 268 |
| | | R:AGGGTTTTCCCAGTCACGCTGGCGAATGGTGAGCAGAC | |
| | Shigella | F:AGGGTTTTCCCAGTCACGATATTCMAGCATCTRACAGCAATAG | 657 |
| | | R:GAGCGGATAACAATTTCACACGGCAAATGATACTTGACGACTTTCT | |
| | C. difficile | F:AGGGTTTTCCCAGTCACGAATGATRCCACAAGAYGATTTAGT | 594 |
| | | R:GAGCGGATAACAATTTCACACAAAGAGAYAATGCATAWGTTCCTCC | |
| | C. jejuni | F:AGGGTTTTCCCAGTCACGCATCTTGAGTTGCTCCATAAGATAT | 445 |
| | | R:GAGCGGATAACAATTTCACACGTGCAACTAAACAAGATACTTTTGCTC | |
| | Y. enterocolitica | F:GAGCGGATAACAATTTCACACTGGGTATACATTGGATAATGACCCT | 336 |
| | | R:AGGGTTTTCCCAGTCACGCGTATGCCATTGACGTCTTACTTG | |
| | EPEC | F:AGGGTTTTCCCAGTCACGTCYGAAAGCGAAATGMTGAAGG | 492 |
| | | R:GAGCGGATAACAATTTCACACCCGACCTGACCAAATGCCA | |
| | ETEC | F:AGGGTTTTCCCAGTCACGTATGATCACGCGAGAGGAACAC | 529 |
| | | R:GAGCGGATAACAATTTCACACCTGGGTCTCCTCATTACAAGTATCA | |
| | EAEC | F:AGGGTTTTCCCAGTCACGGATACAACCCCTGTCTGGAAAT | 608 |
| | | R:GAGCGGATAACAATTTCACACCTAATACAGAATCGTCAGCATCAGC | |
| | EIEC | F:AGGGTTTTCCCAGTCACGGTTCCGCCTCGAAATTCTGG | 340 |
| | | R:GAGCGGATAACAATTTCACACCTCTTCACGGCTTCTGACC | |
| | EHEC | F:AGGGTTTTCCCAGTCACGCATATATCTCAGGGRACCACATC | 387 |
| | | F:AGGGTTTTCCCAGTCACGCATATATCTCAGGGRGCTACATC | |
| | | R:GAGCGGATAACAATTTCACACAACTCCATTAACGCCAGATATGATG | |
| | E. coli O157 | F:GCTCTGCGGTCCTAGTTAGAATTG | 218 |
| | | R:TTGGCATGACGTTATAGGCTACAA | |
| Viruses | Norovirus | F:AGGGTTTTCCCAGTCACGGTACCAGAGGKCAACAAYG | 648 |
| | | R:GAGCGGATAACAATTTCACACGATTCAACWGWDGGTGGCAC | |
| | Rotavirus A | F:AGGGTTTTCCCAGTCACGCACGAATGAAYGCTAAAGTYAAAGC | 777 |
| | | R:GAGCGGATAACAATTTCACACTTCTGGYAADGTYGGCATAAA | |
| | Rotavirus B | F:AGGGTTTTCCCAGTCACGATTCTCCCATGGCAGGTGGC | 709 |
| | | R:GAGCGGATAACAATTTCACACCTCTAGACCYGTATATGAAGCAAGTG | |
| | Rotavirus C | F:AGGGTTTTCCCAGTCACGGAATACACTGATATATACATGGACCC | 615 |
| | | R:GAGCGGATAACAATTTCACACGTTAAAGGATCTGGAGATTTAAACCA | |
| | Adenovirus | F:AGGGTTTTCCCAGTCACGACGGGCACTCTTCGCCTTC | 524/569 |
| | | R:GAGCGGATAACAATTTCACACCAATTCTGAGTTGAAGTTGGTTGT | |
| | Astrovirus | F:AGGGTTTTCCCAGTCACGCAGAAGAGCAACTCCATCGC | 445 |
| | | R:GAGCGGATAACAATTTCACACAGTRCTYCCAGTAGCRTCCTTAAC | |
The highlighted base corresponds to degenerate bases as follows: K(G/T), W(A/T), Y(C/T), R(A/G) , D(G/T/C).
