# Supplementary material for: Clinical application of a multiplex genetic pathogen detection system remaps the aetiology of diarrhoeal infections in Shanghai
Source: Gut Pathog. 2018 Sep 11;10:37. doi: 10.1186/s13099-018-0264-7 (PMC6134694; doi:10.1186/s13099-018-0264-7)
Supplement: Supplementary file 3 — Additional file 3: Figure S1. Primers designed for DP-HMGS effectively detect DPs in individual PCRs. In the DP-HMGS assay reaction system with all DP signature primers present, the nucleic acid templates for each of the pathogens were tested individually. Each class of diarrhoeal pathogens was successfully detected by DP-HMGS. The specific peaks individually appeared at 209 bp for C. jejuni (A), 136 bp for Shigella (B), 202 bp for C. difficile (C), 159 bp for HASV (D), 226 bp for norovirus (E), 190 bp for ETEC (F), 152 bp for EHEC (G), 251 bp for EPEC (H), 292 bp for EAEC (I), 129 bp for EIEC (J), 308 bp for rotavirus (K), 120 bp for S. enteritidis (L), 113 bp for S. typhimurium (M), 196 bp for Vibrio (N), 145 bp for HADV (O), 165 bp for Y. enterocolitica (P) and 218 bp for E. coli O157 (Q). Notably, the peaks in A-Q at 313 bp were for the IC, and all gene targets were specifically amplified without non-specific amplification by individual PCR assays. [file 13099_2018_264_MOESM3_ESM.ppt]

## Slide 1
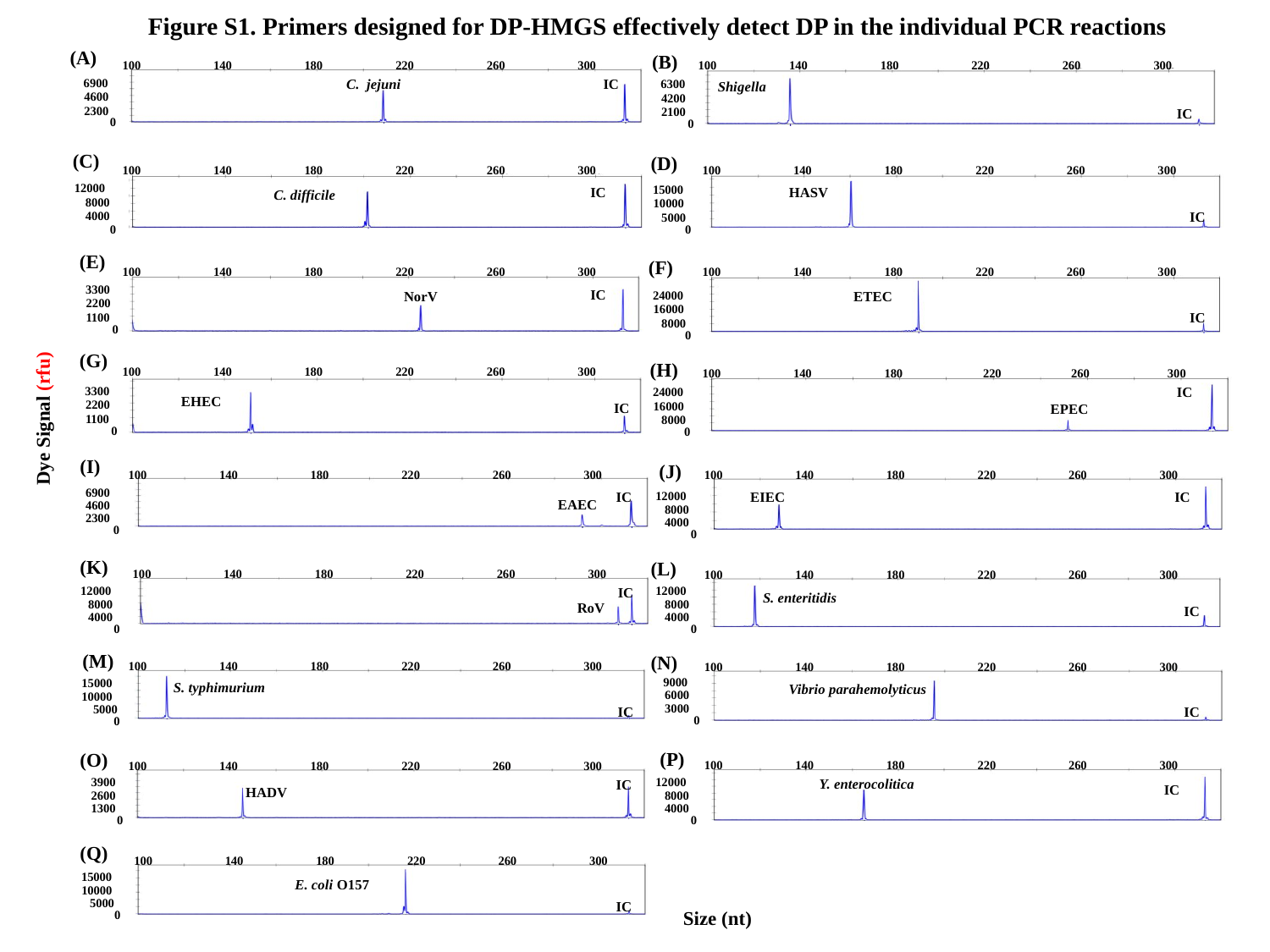

Figure S1. Primers designed for DP-HMGS effectively detect DP in the individual PCR reactions
(A)
(B)
100
140
180
220
260
300
100
140
180
220
260
300
6900
C. jejuni
IC
6300
Shigella
4600
4200
2300
2100
IC
0
0
(C)
(D)
100
140
180
220
260
300
100
140
180
220
260
300
12000
15000
IC
HASV
C. difficile
8000
10000
IC
4000
5000
 0
 0
(E)
(F)
100
140
180
220
260
300
100
140
180
220
260
300
3300
IC
NorV
24000
ETEC
Dye Signal (rfu)
2200
16000
IC
1100
8000
 0
 0
(G)
(H)
100
140
180
220
260
300
100
140
180
220
260
300
3300
IC
24000
EHEC
2200
16000
IC
EPEC
1100
8000
0
 0
(I)
(J)
100
140
180
220
260
300
100
140
180
220
260
300
6900
IC
EIEC
IC
12000
EAEC
4600
8000
2300
4000
0
 0
(K)
(L)
100
140
180
220
260
300
100
140
180
220
260
300
12000
IC
12000
S. enteritidis
8000
8000
RoV
IC
4000
4000
 0
 0
(M)
(N)
100
140
180
220
260
300
100
140
180
220
260
300
 9000
15000
S. typhimurium
Vibrio parahemolyticus
6000
10000
3000
5000
IC
IC
 0
 0
(P)
(O)
100
140
180
220
260
300
100
140
180
220
260
300
3900
12000
Y. enterocolitica
IC
IC
HADV
2600
8000
1300
4000
0
 0
(Q)
100
140
180
220
260
300
15000
E. coli O157
10000
5000
IC
Size (nt)
 0
