# Supplementary material for: Clinical application of a multiplex genetic pathogen detection system remaps the aetiology of diarrhoeal infections in Shanghai
Source: Gut Pathog. 2018 Sep 11;10:37. doi: 10.1186/s13099-018-0264-7 (PMC6134694; doi:10.1186/s13099-018-0264-7)
Supplement: Supplementary file 4 — Additional file 4: Figure S2. The DP-HMGS assay was highly specific for DP identification. (A) In the positive control, DP-HMGS detection of DP was conducted by simultaneous detection of the plasmid templates shown here for 17 pathogens and 3 quality controls. All the targets (from left to right: hum_RNA, S. typhimurium, S. enteritidis, EIEC, Shigella, HADV, EHEC, HASV, Y. enterocolitica, ETEC, Vibrio, C. difficile, C. jejuni, E. coli O157, norovirus, hum_DNA, EPEC, EAEC, rotavirus, IC) could be specifically detected by DP-HMGS. (B–H) DP-HMGS assay results obtained using 7 individual plasmid templates from H. pylori, E. coli DH5α, P. aeruginosa, S. aureus, P. shigelloides, sapovirus, and non-pathogenic E. coli showed no pathogen-specific peak. (I) DP-HMGS assay results obtained by using ddH2O as a DNA template showed no pathogen-specific peak. DP-specific genes produced signals only in the positive controls, not in the negative control, which consisted of pathogens that could potentially interfere with specific pathogen detection. Notably, the peaks in B-H at 313 bp were for the IC. [file 13099_2018_264_MOESM4_ESM.ppt]

## Slide 1
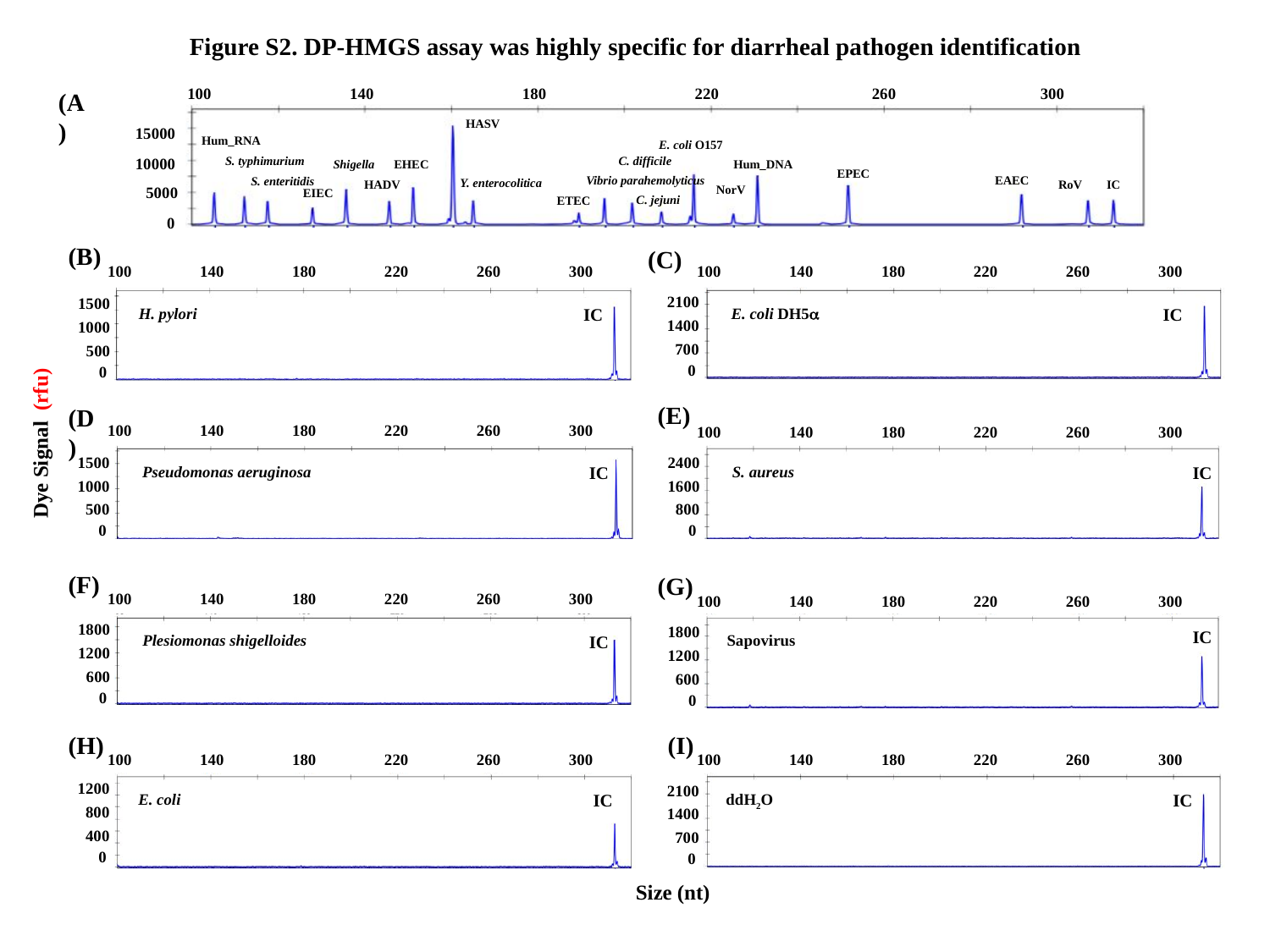

Figure S2. DP-HMGS assay was highly specific for diarrheal pathogen identification
100
140
180
220
260
300
(A)
HASV
15000
Hum_RNA
E. coli O157
10000
S. typhimurium
C. difficile
Shigella
EHEC
Hum_DNA
EPEC
EAEC
Vibrio parahemolyticus
S. enteritidis
Y. enterocolitica
HADV
RoV
IC
NorV
5000
EIEC
C. jejuni
ETEC
0
(B)
(C)
100
140
180
220
260
300
100
140
180
220
260
300
Dye Signal (rfu)
2100
1500
H. pylori
IC
E. coli DH5α
IC
1400
1000
 700
 500
0
0
(E)
(D)
100
140
180
220
260
300
100
140
180
220
260
300
1500
2400
Pseudomonas aeruginosa
IC
 S. aureus
IC
1000
1600
 500
 800
0
0
(F)
(G)
100
140
180
220
260
300
100
140
180
220
260
300
1800
1800
IC
Plesiomonas shigelloides
Sapovirus
IC
1200
1200
 600
 600
0
0
(H)
(I)
100
140
180
220
260
300
100
140
180
220
260
300
1200
2100
IC
IC
E. coli
ddH2O
 800
1400
 400
 700
0
0
Size (nt)
