# Supplementary material for: Clinical application of a multiplex genetic pathogen detection system remaps the aetiology of diarrhoeal infections in Shanghai
Source: Gut Pathog. 2018 Sep 11;10:37. doi: 10.1186/s13099-018-0264-7 (PMC6134694; doi:10.1186/s13099-018-0264-7)
Supplement: Supplementary file 5 — Additional file 5: Figure S3. DP-HMGS could directly detect specific microbial signatures in faecal specimens. (A) The human faecal specimen with a specific DP infection produced a C. difficile-specific peak at 202 bp, as well as specific peaks for the human internal RNA control, human internal DNA control and internal positive test control at 106 bp, 233 bp and 313 bp, respectively. (B) The faecal specimens from non-infectious diarrhoea patients produced only the specific peaks of Hum_RNA, Hum_DNA and IC at 106 bp, 233 bp and 313 bp, respectively. (C) ddH2O showed only the specific peak for the IC at 313 bp. [file 13099_2018_264_MOESM5_ESM.ppt]

## Slide 1
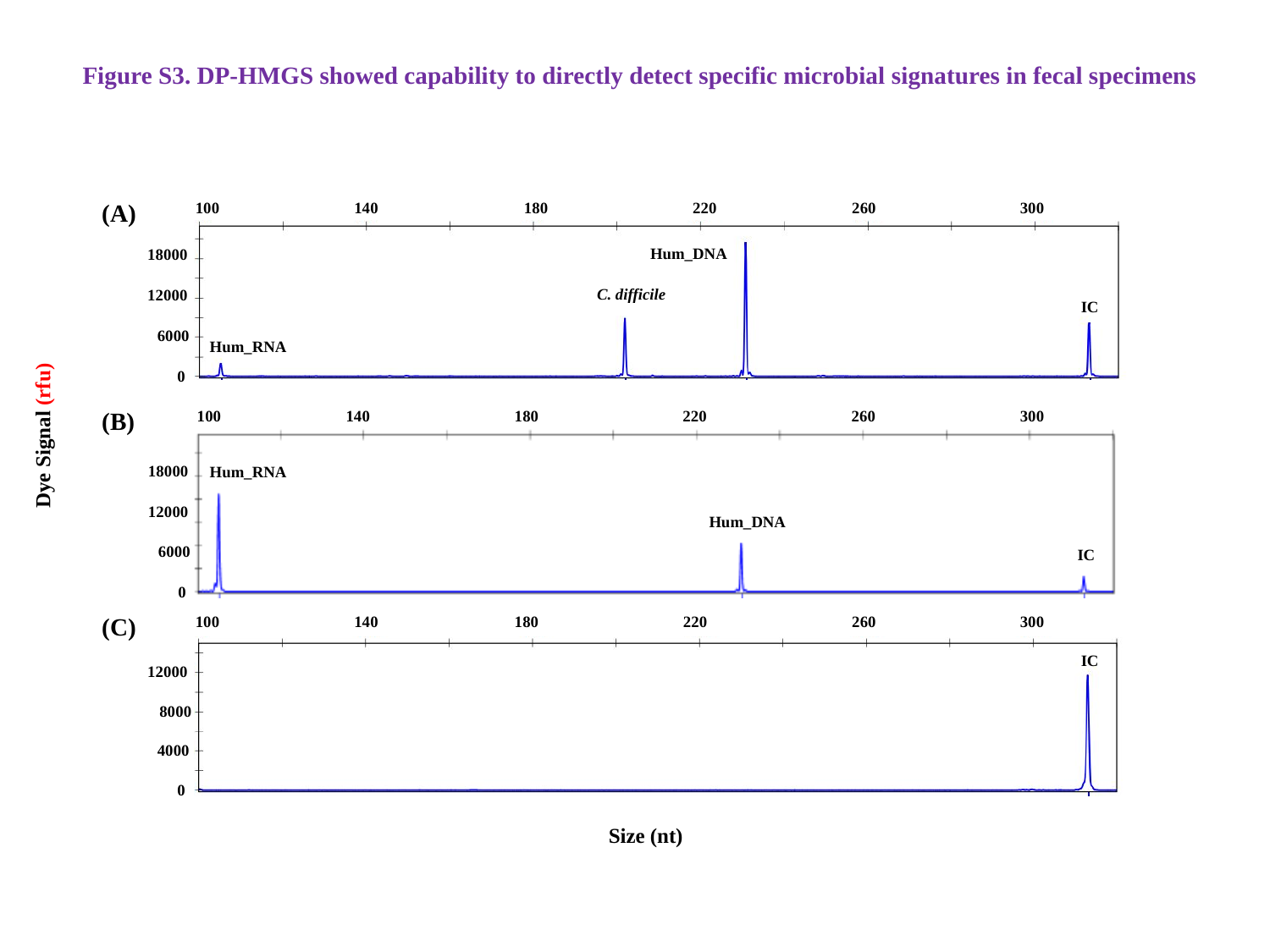

Figure S3. DP-HMGS showed capability to directly detect specific microbial signatures in fecal specimens
(A)
100
140
180
220
260
300
Hum_DNA
18000
C. difficile
12000
IC
Dye Signal (rfu)
6000
Hum_RNA
0
(B)
100
140
180
220
260
300
18000
Hum_RNA
12000
Hum_DNA
6000
IC
0
(C)
100
140
180
220
260
300
IC
12000
 8000
4000
0
Size (nt)
