# Supplementary material for: Clinical application of a multiplex genetic pathogen detection system remaps the aetiology of diarrhoeal infections in Shanghai
Source: Gut Pathog. 2018 Sep 11;10:37. doi: 10.1186/s13099-018-0264-7 (PMC6134694; doi:10.1186/s13099-018-0264-7)
Supplement: Supplementary file 6 — Additional file 6: Table S3. Frequency of the combinations of polymicrobial infections. The combinations and the percentages of the most common polymicrobial DP infections include double infections, triple infections, multi-bacterial infections and bacterial/viral polymicrobial infections. [file 13099_2018_264_MOESM6_ESM.ppt]

## Slide 1
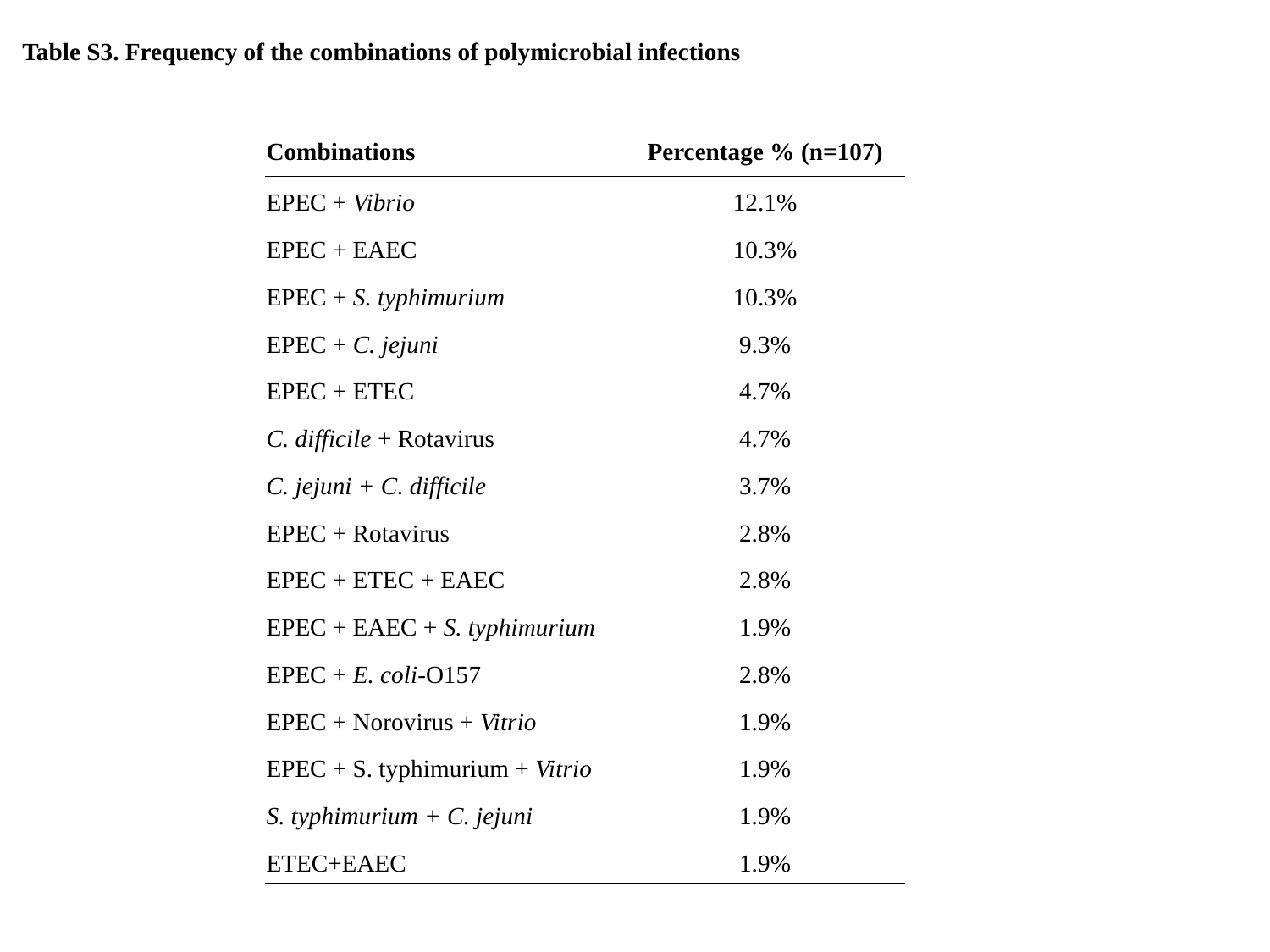

Table S3. Frequency of the combinations of polymicrobial infections
| Combinations | Percentage % (n=107) |
| --- | --- |
| EPEC + Vibrio | 12.1% |
| EPEC + EAEC | 10.3% |
| EPEC + S. typhimurium | 10.3% |
| EPEC + C. jejuni | 9.3% |
| EPEC + ETEC | 4.7% |
| C. difficile + Rotavirus | 4.7% |
| C. jejuni + C. difficile | 3.7% |
| EPEC + Rotavirus | 2.8% |
| EPEC + ETEC + EAEC | 2.8% |
| EPEC + EAEC + S. typhimurium | 1.9% |
| EPEC + E. coli-O157 | 2.8% |
| EPEC + Norovirus + Vitrio | 1.9% |
| EPEC + S. typhimurium + Vitrio | 1.9% |
| S. typhimurium + C. jejuni | 1.9% |
| ETEC+EAEC | 1.9% |
